# Intramembrane ionic protein–lipid interaction regulates integrin structure and function

**DOI:** 10.1371/journal.pbio.2006525

**Published:** 2018-11-14

**Authors:** Jun Guo, Youhua Zhang, Hua Li, Huiying Chu, Qinshu Wang, Shutan Jiang, Yan Li, Hongbin Shen, Guohui Li, Jianfeng Chen, Chenqi Xu

**Affiliations:** 1 State Key Laboratory of Molecular Biology, Shanghai Science Research Center, CAS Center for Excellence in Molecular Cell Science, Shanghai Institute of Biochemistry and Cell Biology, Chinese Academy of Sciences, University of Chinese Academy of Sciences, Shanghai, China; 2 State Key Laboratory of Cell Biology, CAS Center for Excellence in Molecular Cell Science, Shanghai Institute of Biochemistry and Cell Biology, Chinese Academy of Sciences, Shanghai, China; 3 Department of Pathology, Shanghai Tenth People's Hospital, Tongji University School of Medicine, Shanghai, China; 4 Laboratory of Molecular Modeling and Design, State Key Laboratory of Molecular Reaction Dynamics, Dalian Institute of Chemical Physics, Chinese Academy of Science, Dalian, Liaoning, China; 5 School of Life Science and Technology, ShanghaiTech University, Shanghai, China; 6 Institute of Image Processing and Pattern Recognition, Shanghai Jiaotong University, Shanghai, China; University of Pennsylvania Perelman School of Medicine, United States of America

## Abstract

Protein transmembrane domains (TMDs) are generally hydrophobic, but our bioinformatics analysis shows that many TMDs contain basic residues at terminal regions. Physiological functions of these membrane-snorkeling basic residues are largely unclear. Here, we show that a membrane-snorkeling Lys residue in integrin αLβ2 (also known as lymphocyte function-associated antigen 1 [LFA-1]) regulates transmembrane heterodimer formation and integrin adhesion through ionic interplay with acidic phospholipids and calcium ions (Ca^2+^) in T cells. The amino group of the conserved Lys ionically interacts with the phosphate group of acidic phospholipids to stabilize αLβ2 transmembrane association, thus keeping the integrin at low-affinity conformation. Intracellular Ca^2+^ uses its charge to directly disrupt this ionic interaction, leading to the transmembrane separation and the subsequent extracellular domain extension to increase adhesion activity. This Ca^2+^-mediated regulation is independent on the canonical Ca^2+^ signaling or integrin inside-out signaling. Our work therefore showcases the importance of intramembrane ionic protein–lipid interaction and provides a new mechanism of integrin activation.

## Author summary

Integrin αLβ2 is the major integrin in T cells and plays a vital role in regulating T-cell activation, adhesion, and migration. The transmembrane association of αL and β2 is crucial for maintaining the integrin at low-affinity conformation. Here, we find that the conserved basic residue (K702) in the transmembrane domain of β2 contributes to transmembrane association through ternary ionic interaction with acidic phospholipid and αL cytoplasmic residue. Upon T-cell activation, influxed calcium ions (Ca^2+^) can directly disrupt the ionic K702–lipid interaction through its positive charges, which leads to transmembrane separation and subsequent extracellular domain extension to switch αLβ2 to high-affinity conformation. This Ca^2+^-mediated regulation is through the modulation of the ionic Lys–lipid interaction but not through the canonical Ca^2+^ signaling or integrin inside-out signaling. Our study thus reports a new regulatory mechanism of integrin activation and showcases the importance of intramembrane ionic protein–lipid interaction. This finding might have general relevance, as bioinformatics analysis shows the presence of membrane-snorkeling basic residue is a common feature of transmembrane proteins.

## Introduction

Cell membrane contains two distinct lipid bilayers. For the plasma membrane of mammalian cells, the outer leaflet is enriched of sphingolipid, cholesterol, and phosphatidylcholine, whereas the inner leaflet comprises of acidic phospholipids such as phosphatidylserine and phosphatidylinositides [1]. Negatively charged acidic phospholipids can ionically interact with positively charged protein domains or sequences to regulate protein structure and function [2, 3]. It has been well demonstrated that acidic phospholipids are able to bind to juxtamembrane polybasic sequences of transmembrane proteins and membrane-anchored proteins to regulate protein signaling [4–8], clustering [9, 10], and localization [11, 12]. Intriguingly, recent evidences suggest that the intramembrane basic residue close to the transmembrane domain (TMD) and cytoplasmic domain (CD) border could also ionically interact with acidic phospholipids [13, 14]. However, it is still unclear whether the “membrane-snorkeling” basic residue is a general feature of transmembrane domains. Particularly, it is important to investigate how membrane protein activity is regulated by intramembrane ionic protein–lipid interaction under physiological conditions.

Here, we first performed a bioinformatics analysis of single-span membrane proteins from yeast and human and demonstrate that the membrane-snorkeling basic residue is an evolutionarily conserved feature of transmembrane proteins. We chose integrin αLβ2, a key adhesion molecule in T cells, to reveal the importance of the intramembrane ionic protein–lipid interaction in regulating membrane protein structure and function. Though the antagonist of αLβ2 has been clinically approved for treating local inflammation [15], the regulatory mechanism of αLβ2 activation is not fully understood. Combining nuclear magnetic resonance (NMR) spectroscopy, molecular dynamics (MD) simulations, fluorescence resonance energy transfer (FRET), and flow chamber assays, we find that the membrane-snorkeling Lys702 in β2 chain acts as a gatekeeper for αLβ2 activity through ionic interaction with the phosphate group of acidic phospholipids. Moreover, the intracellular Ca^2+^ directly disrupts the intramembrane Lys–lipid interaction to activate αLβ2 and then promotes T-cell adhesion. Our results uncover a novel mechanism of protein–lipid interaction, which might have general application in membrane protein signaling and also shed new light on modulating T-cell migration and adhesion in various disease contexts such as cancer and autoimmune diseases.

## Results

### General relevance of membrane-snorkeling basic residue

Membrane-spanning proteins typically have hydrophobic residues in their transmembrane domains to facilitate hydrophobic interactions with lipid acyl chains. Presence of charged residues at TMD terminus, however, is tolerable because the lipid headgroup region is hydrophilic.

**Fig 1:**
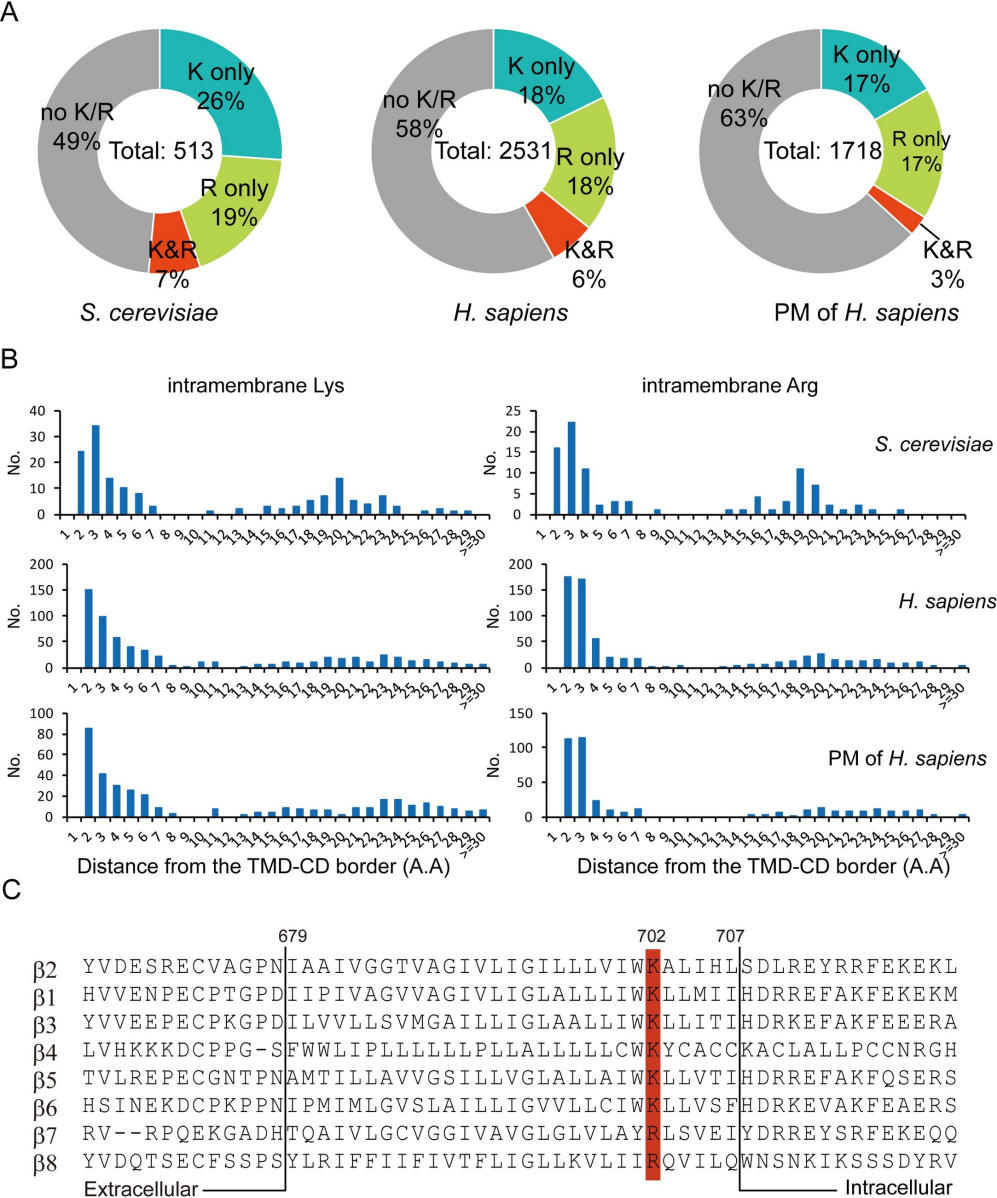
Membrane-snorkeling basic residues in transmembrane domains. (A) Percentage distribution of single-span transmembrane proteins containing intramembrane Lys or Arg, both Lys and Arg, or no Lys or Arg. Data sets include single-span transmembrane proteins from yeast (*Saccharomyces cerevisiae*), human (*Homo sapiens*), as well as human single-span transmembrane proteins located at the PM (of *H*. *sapiens*) [28]. (B) Location of intramembrane basic residue. The x-axis indicates the distance of intramembrane basic residue from the TMD/CD border, and “1” means the first TMD residue on the border. Supporting data are compiled in S1 Data. The underlying data of panel A and B can be found in http://dx.doi.org/10.17632/tg2622h9dd.1. (C) Sequence alignment of eight human integrin β subunit TMDs. The conserved intramembrane Lys/Arg are highlighted in red. Residues are numbered according to the human β2 sequence. Lys, lysine; Arg, arginine; CD, cytoplasmic domain; TMD, transmembrane domain; PM, plasma membrane.

To investigate whether containing basic residues is a general feature for transmembrane domains, we analyzed the TMD sequences of single-span transmembrane proteins from yeast and human. More than 40% of the TMDs from both yeast and human contain lysine (Lys) or arginine (Arg) (Fig 1A). These basic residues mainly localize close to the border between TMD and CD (Fig 1B). More specifically, we analyzed the human single-span transmembrane proteins located at the plasma membrane and confirmed the high frequency of membrane-snorkeling basic residues (Fig 1A and 1B). Within the list, we found that all eight members of human integrin β subunits contain an intramembrane Lys/Arg residue at the position that is six residues away from the TMD/CD border (Fig 1C). Integrins are α/β heterodimeric adhesion molecules that mediate cell–cell, cell–matrix, and cell–pathogen interactions [16–20]. Of them, αLβ2 is the major integrin in T cells that regulates T-cell activation, effector function, and differentiation [21–27]. We therefore studied the activation mechanism of αLβ2.

### The membrane-snorkeling Lys stabilizes αLβ2 dimer

Integrin α and β transmembrane domains form dynamic association that keeps integrin at low-affinity conformation [13, 29, 30]. We applied solution NMR to study the role of the membrane-snorkeling Lys in αLβ2 transmembrane interaction.

**Fig 2:**
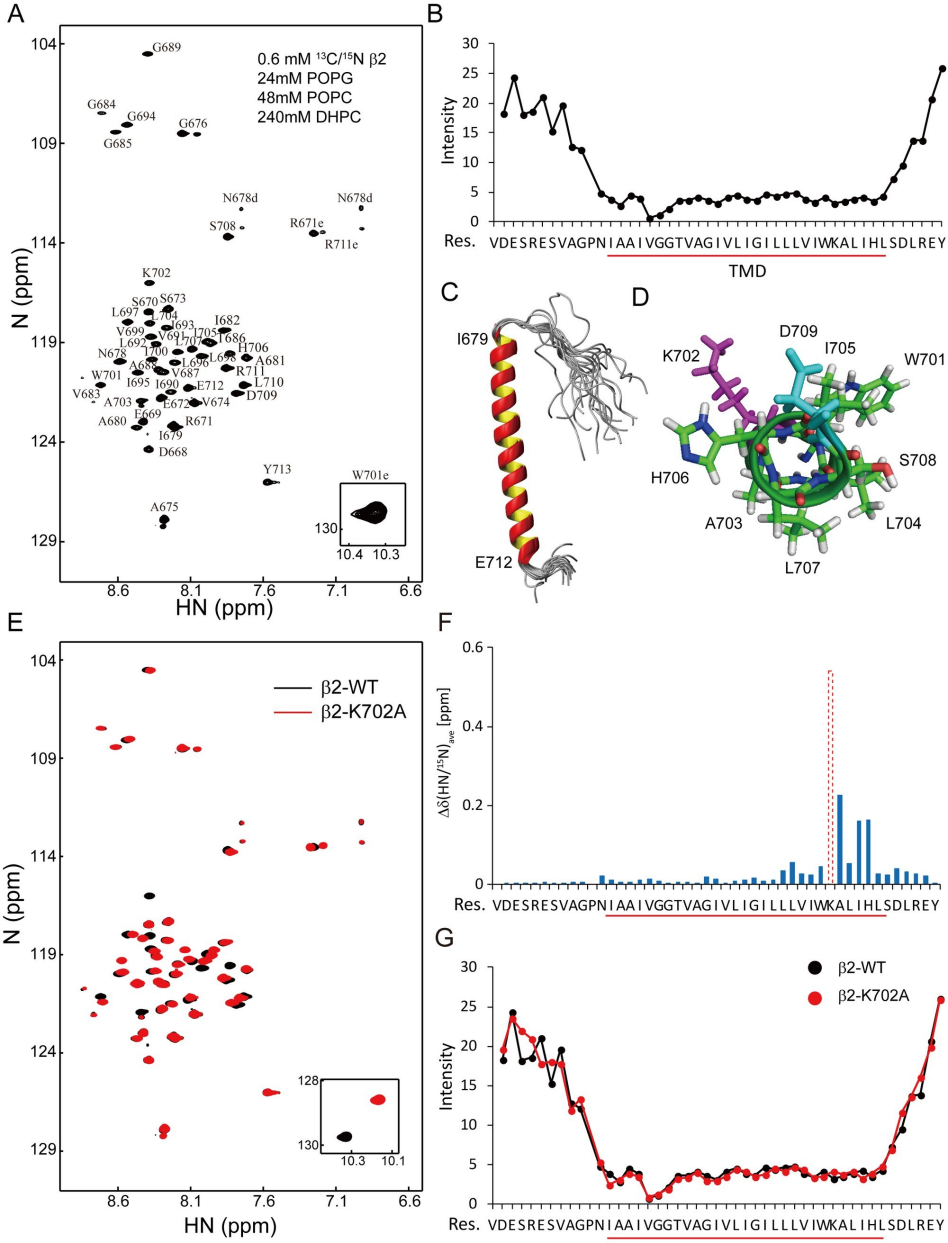
Structural feature of β2 monomer. Human β2-WT or β2-K702A mutant construct was reconstituted in the mixture lipid bicelles (33% POPG, 67% POPC). NMR experiments were performed to acquire structural information of β2 monomer. The NMR sample of β2 monomer (β2-WT or β2-K702A) contained 0.6 mM ^13^C/^15^N-labled β2 TMD peptide, 20 mM Bis-Tris (pH 6.7), 240 mM DHPC, 24 mM POPG, and 48 mM POPC. (A) Backbone assignments of the ^1^H-^15^N TROSY-HSQC spectrum of the β2-WT construct. (B) Peak intensities of backbone amide signals of the β2-WT construct measured in (A). The transmembrane domain (I679-L707) is underlined. (C) Backbone superimposition of the 20 NMR structures with the lowest CYANA target function values of the β2-WT construct. The α-helix starts from the N-terminal residue I679 of the TMD and ends at E712 in the CD. (D) Local helical structure of the membrane-snorkeling K702. (E–G) Comparison of ^1^H-^15^N TROSY-HSQC spectra of β2-WT and β2-K702A mutant. Spectra are superimposed in (E). The CSP caused by the K702A mutation is shown in (F). Comparison of peak intensities of β2-WT and K702A mutant is shown in (G). The underlying data of panel B, F, and G can be found in http://dx.doi.org/10.17632/tg2622h9dd.1. ^1^H, hydrogen-1; ^15^N, nitrogen-15; ^13^C, carbon-13; CSP, chemical shift perturbation; NMR, nuclear magnetic resonance; TROSY, transverse relaxation-optimized spectroscopy; HSQC, heteronuclear single quantum coherence; CYANA, combined assignment and dynamics algorithm for NMR applications; DHPC, 1,2-dihexanoyl-sn-glycero-3-phosphocholine; POPC, 1-palmitoyl-2-oleoyl-glycero-3-phosphocholine; POPG, 1-palmitoyl-2-oleoyl-sn-glycero-3-phospho-(1'-rac-glycerol); TMD, transmembrane domain; CD, cytoplasmic domain; WT, wild type

We first reconstituted a human β2 construct that contains the TMD, short extracellular domain (ED), and CD into a lipid bicelle system containing both zwitterionic phospholipid 1-palmitoyl-2-oleoyl-glycero-3-phosphocholine (POPC) and acidic phospholipid 1-palmitoyl-2-oleoyl-sn-glycero-3-phospho-(1'-rac-glycerol) (POPG) (33% POPG, 67% POPC). The well-dispersed hydrogen-1, nitrogen-15 (^1^H-^15^N) transverse relaxation-optimized spectroscopy (TROSY) spectrum indicated successful folding of the β2 TMD peptide. Chemical shifts of amide groups were assigned for all residues except the fast-tumbling N-terminal V667 (Fig 2A). Compared with the extracellular and intracellular regions, the hydrophobic I679-L707 region showed much lower signal intensity, which might be caused by the slow tumbling of this region within the membrane bilayer (Fig 2B). We defined this hydrophobic region as the TMD, which is consistent with the definition in other integrin β chains [31]. The β2 monomer structure showed that the TMD formed nearly a straight α-helix that extended till E712 in the CD (Fig 2C and S1 Table). To further confirm the position of K702, we used a membrane-incorporating paramagnetic probe 16-doxyl stearic acid (16-DSA), with the paramagnetic spin label at the C16 position. Since the paramagnetic relaxation enhancement (PRE) effect negatively correlates with the distance between the residue and paramagnetic spin label, the core region (V683-L697) of TMD showed dramatic intensity changes while the N-terminus (A680, A681, I682) and C-terminus (I705, H706, L707) of TMD showed moderate change. The fact that the PRE effect of K702 is between that of the most solvent exposed residues (such as S673, R671, D709) and the membrane-core region (V683-L697) (S1 Fig) confirms that K702 is a membrane-snorkeling residue located in the lipid headgroup region. The membrane snorkeling of the corresponding Lys in β3 (K716) and β1 (K752) has also been reported previously [31–33]. The K702 sidechain pointed to a direction different from those of the neighboring hydrophobic residues W701 and I705 that might involve in dimerization according to the αIIbβ3 structure [31] (Fig 2D). Mutating K702 to alanine (Ala, A) did not affect the signal intensities of the TMD residues but induced substantial chemical shift changes of the C-terminal residues (Fig 2E–2G), which implied that K702 might not affect overall transmembrane topology but regulate local conformation instead.

**Fig 3:**
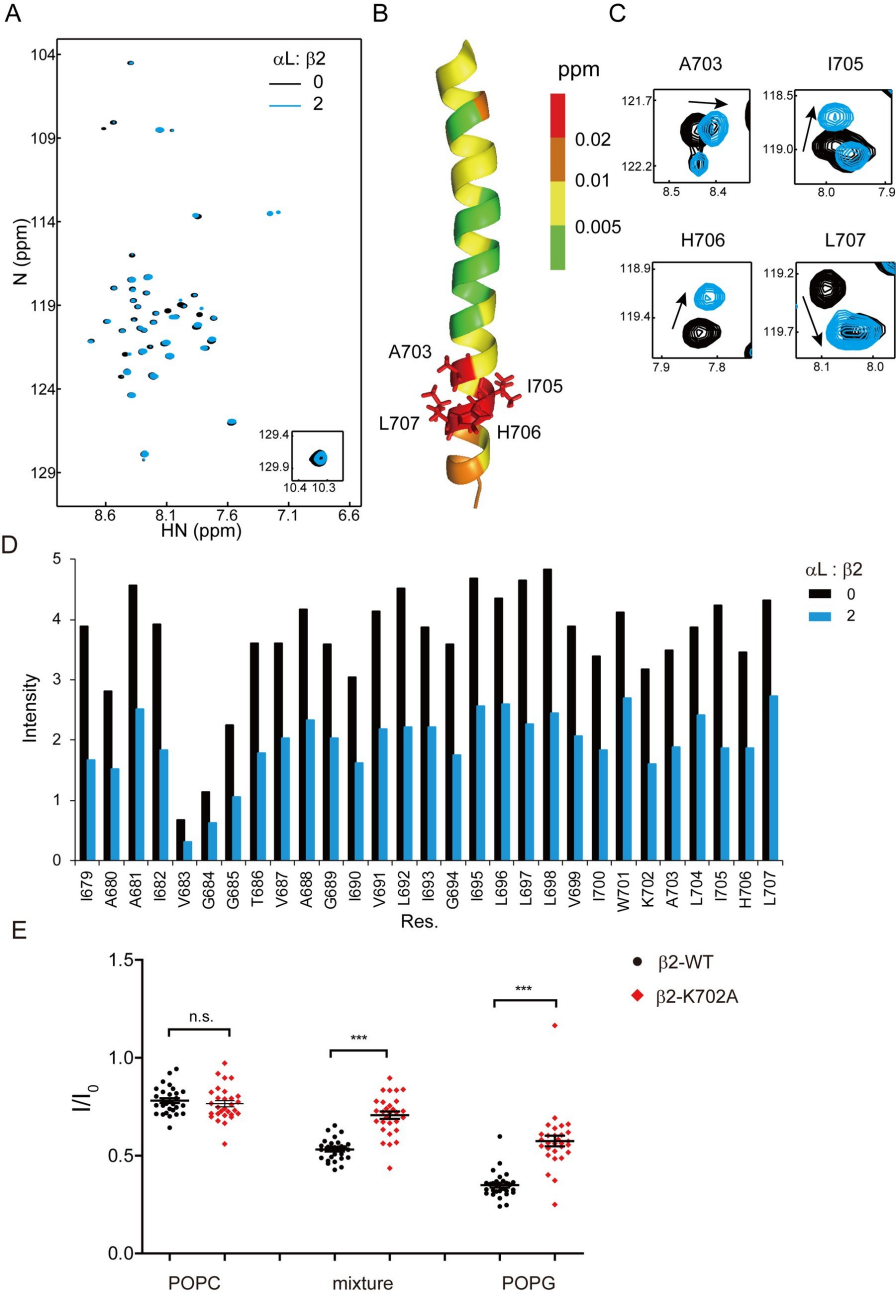
Membrane-snorkeling Lys and acidic phospholipids stabilize the dynamic αLβ2 transmembrane association. (A–D) ^13^C/^15^N-labeled β2-WT was mixed with unlabeled αL in the mixture lipid bicelles to form heterodimer. The β2-WT monomer contained 0.6 mM ^13^C/^15^N-labeled β2 TMD peptide, 20 mM Bis-Tris (pH 6.7), 240 mM DHPC, 24 mM POPG, and 48 mM POPC. In the αLβ2 transmembrane heterodimer sample, an additional 1.2 mM αL peptide was reconstituted into the bicelles. (A) Superimposed ^1^H-^15^N TROSY-HSQC spectra of ^13^C/^15^N-labeled β2-WT in the presence or absence of unlabeled αL. (B) Chemical shift perturbation of β2-WT caused by αL titration is displayed on the β2 monomer structure by color code. (C) Superimposed ^1^H-^15^N TROSY-HSQC spectra of the most affected β2 residues by αL titration. (D) Signal intensity comparison of β2 TMD residues in the presence or absence of αL. (E) Scatter plots of β2-WT/K702A signal intensity reduction upon dimer formation in different lipid bicelles. *I* represents the signal intensity of β2 TMD residue in the dimer sample, while *I*_0_ represents the corresponding one in the monomer sample. Each dot represents a single β2 TMD residue. Three different phospholipid bicelles (72 mM), i.e., POPC, mixture (33% POPG, 67% POPC) and POPG bicelles, were applied to provide membrane environment with different charge properties. The sample condition was the same as (A–D). The underlying data of panel D and E can be found in http://dx.doi.org/10.17632/tg2622h9dd.1. Paired *t* test was used to compare the difference between β2-WT and β2-K702A mutant (*n* = 29 for each group). ***P < 0.001. n.s., not significant; ^1^H, hydrogen-1; ^15^N, nitrogen-15; ^13^C, carbon-13; TROSY, transverse relaxation-optimized spectroscopy; HSQC, heteronuclear single quantum coherence; DHPC, 1,2-dihexanoyl-sn-glycero-3-phosphocholine; POPC, 1-palmitoyl-2-oleoyl-glycero-3-phosphocholine; POPG, 1-palmitoyl-2-oleoyl-sn-glycero-3-phospho-(1'-rac-glycerol); TMD, transmembrane domain; WT, wild type

We next mixed unlabeled αL transmembrane peptide with nitrogen-15 (^15^N)-labeled β2 transmembrane peptide to study the dimerization process. Addition of αL only caused minor chemical shift changes of β2, mainly at the A703-L707 region that is exactly after the membrane-snorkeling K702 (Fig 3A–3C). No new set of resonances was observed. Instead, signal intensity reduction was observed for all β2 TMD residues (Fig 3D and S2A Fig), which reflects the intermediate αLβ2 TMD heterodimer exchange rate [34]. This agrees with a previous paper showing that αLβ2 transmembrane domains display moderate binding affinity [33]. To study whether protein concentration increase could affect signal intensity, we used different concentrations of unlabeled β2 peptide to titrate ^15^N-labeled β2 and found no significant signal intensity reduction (S2B Fig). These results suggest that the signal intensity reduction is mainly caused by the specific αLβ2 heterodimer formation. Such a signal reduction phenomenon is not only observed in our study but also in others [14, 35]. These data suggest that αLβ2 transmembrane association should be relatively weak, and the K702 local region might play an important role in the dimerization. We also checked αLβ2 dimerization at different lipid-to-peptide ratios (ratio ranging from 60:1 to 240:1) or in different sizes of lipid bicelles (*q* values ranging from 0.3 to 0.5) (S3B Fig). In all conditions we tested, only signal reductions but no new peaks were observed (S3 Fig). The condition of small *q* (*q* = 0.3) and intermediate lipid-to-protein ratio (120:1) has been chosen in the later study. These results together suggest that the signal reduction is caused by the specific heterodimer formation rather than condition-specific phenomenon. Thereafter, signal reduction of β2 TMD residues was utilized to indicate αLβ2 dimerization level in the following experiments. Mutation of K702 to Ala obviously impaired αLβ2 dimerization in the mixture lipid bicelles (Fig 3E). We further studied the role of acidic phospholipids in αLβ2 dimerization. Three lipid bicelle systems, i.e., zwitterionic phospholipid bicelles (100% POPC), mixture phospholipid bicelles (33% POPG, 67% POPC), and acidic phospholipid bicelles (100% POPG), were employed for NMR measurements. The change of membrane charge environment significantly affected β2 TMD signals of the αLβ2 dimer (Fig 3E and S4 Fig) but not those of the β2 monomer (S5 Fig). Higher percentage of acidic phospholipids caused more β2 TMD signal reduction, i.e., higher dimerization level, which is consistent with the previous study on αIIbβ3 [36]. The K702A mutation did not affect αLβ2 dimerization in the zwitterionic phospholipid bicelles but substantially impaired dimerization level in the bicelles containing acidic phospholipids (Fig 3E).

Taken together, our data show that the membrane-snorkeling K702 can stabilize αLβ2 transmembrane interaction, and this effect is dependent on acidic phospholipids.

### Ionic interaction between the K702 amino group and the lipid phosphate group

**Fig 4:**
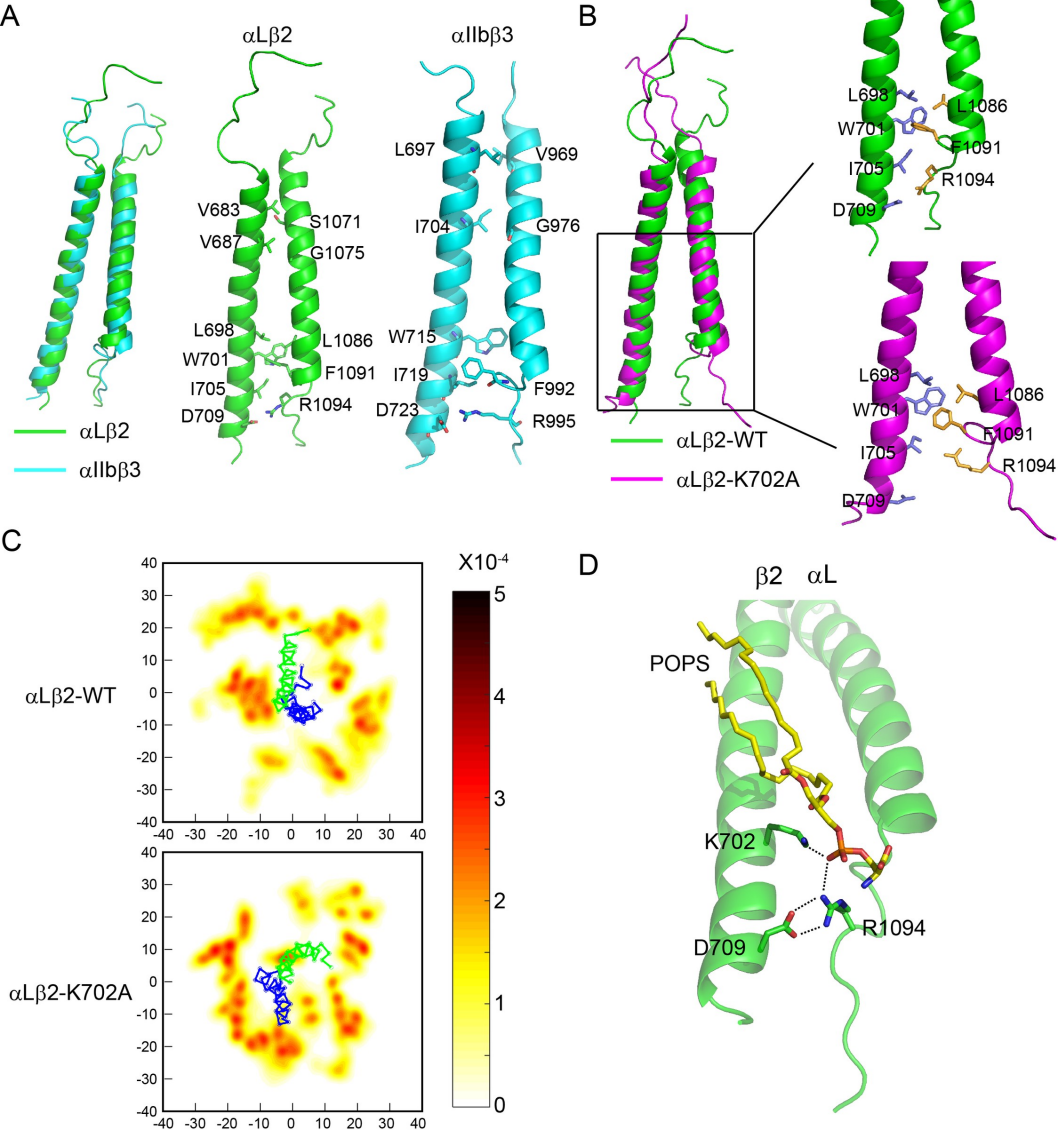
Ionic interaction between the Lys amino group and the lipid phosphate group. Integrin αLβ2 transmembrane association was simulated in a POPS/POPC asymmetric model membrane. (A) Comparison of simulated αLβ2 and αIIbβ3 dimer structures. The sidechains of major residues involved in dimerization are shown in sticks. β chain is displayed on the left and α chain on the right. (B) Comparison of simulated αLβ2-WT and αLβ2-K702A dimer structures in POPS/POPC model membrane. (C) The spatial distribution of POPS around αLβ2-WT or αLβ2-K702A in the membrane inner leaflet is projected onto the membrane (xy) plane. The Cα of αL and β2 chains are also projected (αL shown as blue, and β2 shown as green) to show the possible POPS binding sites. The color bar is set in the range of [0–5 × 10^−4^]. (D) Trimeric interaction among αL, β2, and POPS. The lipid phosphate group simultaneously engages the β2-K702 amino group and the αL-R1094 guanidino group. MD, molecular dynamics; POPC, 1-palmitoyl-2-oleoyl-glycero-3-phosphocholine; POPS, 1-palmitoyl-2-oleoyl-sn-glycero-3-phospho-L-serine; WT, wild type

Since the αLβ2 transmembrane dimer structure is too dynamic to be solved by solution NMR, we applied all-atom MD simulations to study the dynamic association between αL and β2 transmembrane domains. In all of the classical force fields, electrostatic interaction is simply treated, and explicit electronic polarizability is neglected. The condensed-phase polarization, relative to the gas-phase charge distributions, is commonly accounted in an average way by increasing the atomic charges, which remains fixed throughout simulations. The investigation on several ion channels and transporters showed that although the fundamental physical properties could be described using the nonpolarizable models, a more detailed understanding of the conformation-driven super-selectivity depends on improvements in force field models, considering explicit polarizability [37]. Therefore, the MD simulations are performed based on the polarizable atomic multipole-based force field [38]. To mimic the lipid distribution in the plasma membrane, we applied an asymmetric lipid bilayer system, with the outer leaflet containing 100% POPC and the inner leaflet containing 33% POPS and 67% POPC (total 120 POPS/POPC molecules). αL and β2 transmembrane constructs used in the simulations were the same as those in the NMR experiments. Three independent MD simulations were carried out for at least 300 ns on each system, and the snapshots of the last 50 ns in each simulation were used to do further analysis. The conformations sampled from three independent trajectories were used for the clustering analysis [39]. One representative structure was selected from the largest cluster and used in the following analysis. The simulated αLβ2 structure showed a good degree of similarity with the αIIbβ3 NMR structure (Fig 4A). The N-terminal interactions were mainly hydrophobic stacking, while the C-terminal interactions were both hydrophobic stacking (β2-L698/W701/I705 with αL-L1086/F1091) and salt–bridge interactions (β2-D709 and αL-R1094). Mutating β2-K702 to Ala destabilized C-terminal contacts but had little effect on N-terminal contacts (Fig 4B). Analysis of lipid distribution surrounding the dimer showed a clear enrichment of POPS molecules around the C-terminal β2 chain, and such enrichment was impaired in the β2-K702A mutant construct (Fig 4C). We observed that the phosphate group interacted with the amino group of the β2-K702 and, meanwhile, with the guanidino group of αL-R1094 (Fig 4D). The interaction energy data of pairwise atoms confirmed that the lipid PO_4_^−^ group had dominant interaction with the β2-K702-NH_3_^+^ group and αL-R1094-guanidino group (S6 Fig). In summary, MD simulations indicate that the lipid phosphate group simultaneously engages the β2-K702 amino group and the αL-R1094 guanidino group to mediate local contacts and thus stabilizes αLβ2 transmembrane dimer.

### Gatekeeper function of the membrane-snorkeling Lys in restraining αLβ2 activity

**Fig 5:**
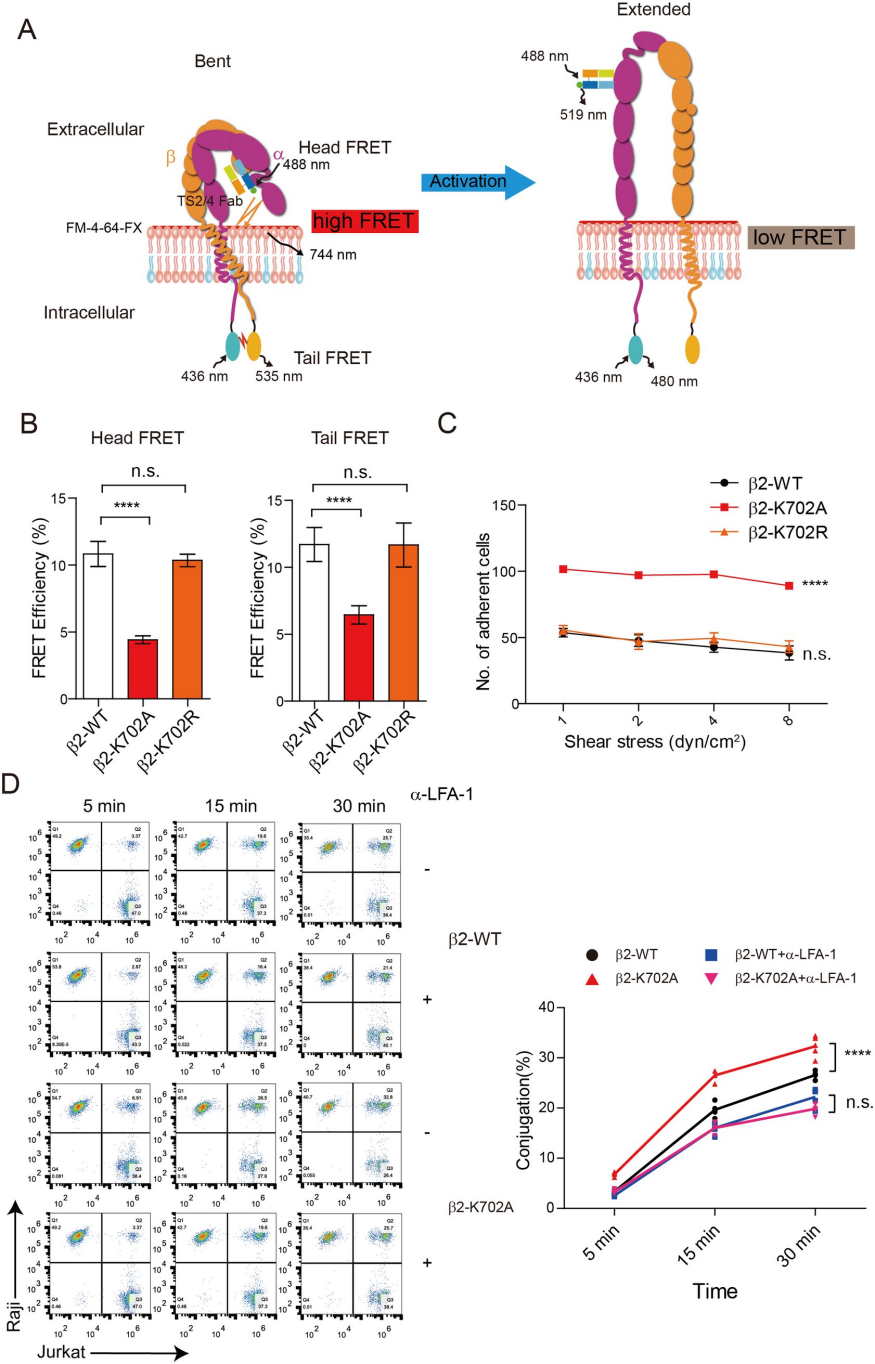
Loss of intramembrane positive charge causes spontaneous activation of αLβ2 in T cells. (A) Schematic representation of the Head and Tail FRET assays that measure the conformation of integrin molecule in live T cells. In the low-affinity integrin conformation (left), extracellular domains are bent toward the plasma membrane, and the α and β transmembrane/cytoplasmic domains are in close proximity. While in the high-affinity conformation (right), the extracellular domains are extended away from the plasma membrane, and the transmembrane/cytoplasmic domains are separated. The Head FRET assay measures distance between the Alexa488-TS 2/4 Fab-labeled αL extracellular domain and the FM-4-64-FX-labeled plasma membrane. The tail FRET measures distance between αL-mTurquoise2 and β2-mCitrine. (B) The Head and Tail FRET of αLβ2 bearing β2-WT, K702A mutant, or K702R mutant. (C) T-cell adhesion to immobilized ICAM-1 substrate was measured by a flow chamber assay. Jurkat T cells expressing β2-WT or K702A mutant were compared (*n* = 3 for each group). Two-way ANOVA was used to analyze the difference. (D) T-cell adhesion to target cells measured by flow cytometry. Jurkat T cells and Raji B cells were labeled with Cell Tracker CFSE and Cell Tracker Deep Red, respectively. To block LFA-1–ICAM-1 interaction, Jurkat T cells were pretreated with 10 μg/ml α-LFA-1 (TS1/18). Representative FACS pictures are shown at the left. The conjugates appear at the right upper corner. Two-way ANOVA was used to compare the differences between β2-WT and β2-K702A in different time points (*n* = 5 for each group). The underlying data of panel B–D can be found in http://dx.doi.org/10.17632/tg2622h9dd.1. Data are representative of three independent experiments and displayed as individual points. ****P < 0.0001. CFSE, 5-(and-6)-Carboxyfluorescein Diacetate, Succinimidyl Ester; FACS, fluorescence activated cell sorting; FRET, florescence resonance energy transfer; ICAM-1, intercellular adhesion molecules 1; LFA-1, lymphocyte function associated antigen 1; n.s., not significant; WT, wild type

Next, we used two types of FRET experiments [40], i.e., the Head FRET and the Tail FRET, to monitor αLβ2 conformational change in live T cells (Fig 5A). The change of integrin from low-affinity to high-affinity states is associated with global conformational rearrangements, including extension of the ED and separation of the α/β cytoplasmic tails. The K702A mutation in β2 resulted in substantial reduction of both Tail and Head FRET efficiencies, indicating integrin activation. In contrast, the K702R mutant that preserves the positive charge showed similar FRET efficiency to the WT (Fig 5B). A flow chamber assay was further applied to determine αLβ2 adhesion to its ligand intercellular adhesion molecule 1 (ICAM-1) under different shear stress. Consistently, the K702A mutation remarkably up-regulated αLβ2 activity while the K702R mutation exerted undetectable influence (Fig 5C). We then used a dual-color flow cytometry assay to assess the conjugation of T cells with ICAM-1–expressing Raji B cells. In line with the above data, the K702A mutation increased the conjugation between T cells and B cells. This increasement was caused by constitutively activation of αLβ2, as the difference disappeared when an LFA-1 antibody but not a CD2 antibody was applied (Fig 5D and S7 Fig).

Collectively, our functional assays show the physiological relevance of β2-K702’s regulation on αLβ2 conformation.

### Ca^2+^ disrupts the ionic Lys–lipid interaction to destabilize αLβ2 dimer

Elevation of intracellular Ca^2+^ concentration is an early hallmark of T-cell activation [41]. More specifically, the major Ca^2+^ channel of T cells, calcium release-activated channels (CRAC), colocalizes with αLβ2 in the immunological synapse to trigger high local Ca^2+^ concentration [42]. Ca^2+^ has been recognized as a master regulator of T-cell adhesion for a long time [43]. Increase of intracellular Ca^2+^ concentration is both necessary and sufficient to induce T-cell stop signals [44, 45]. Ca^2+^ has a strong binding affinity with the lipid phosphate group, and its small hydrodynamic radius makes Ca^2+^ more suitable than magnesium ion (Mg^2+^) to directly bind to the lipid phosphate group [46, 47]. We therefore propose that Ca^2+^ might interfere with the ionic interaction between β2-K702 and acidic phospholipids to regulate αLβ2 conformation and activity.

**Fig 6:**
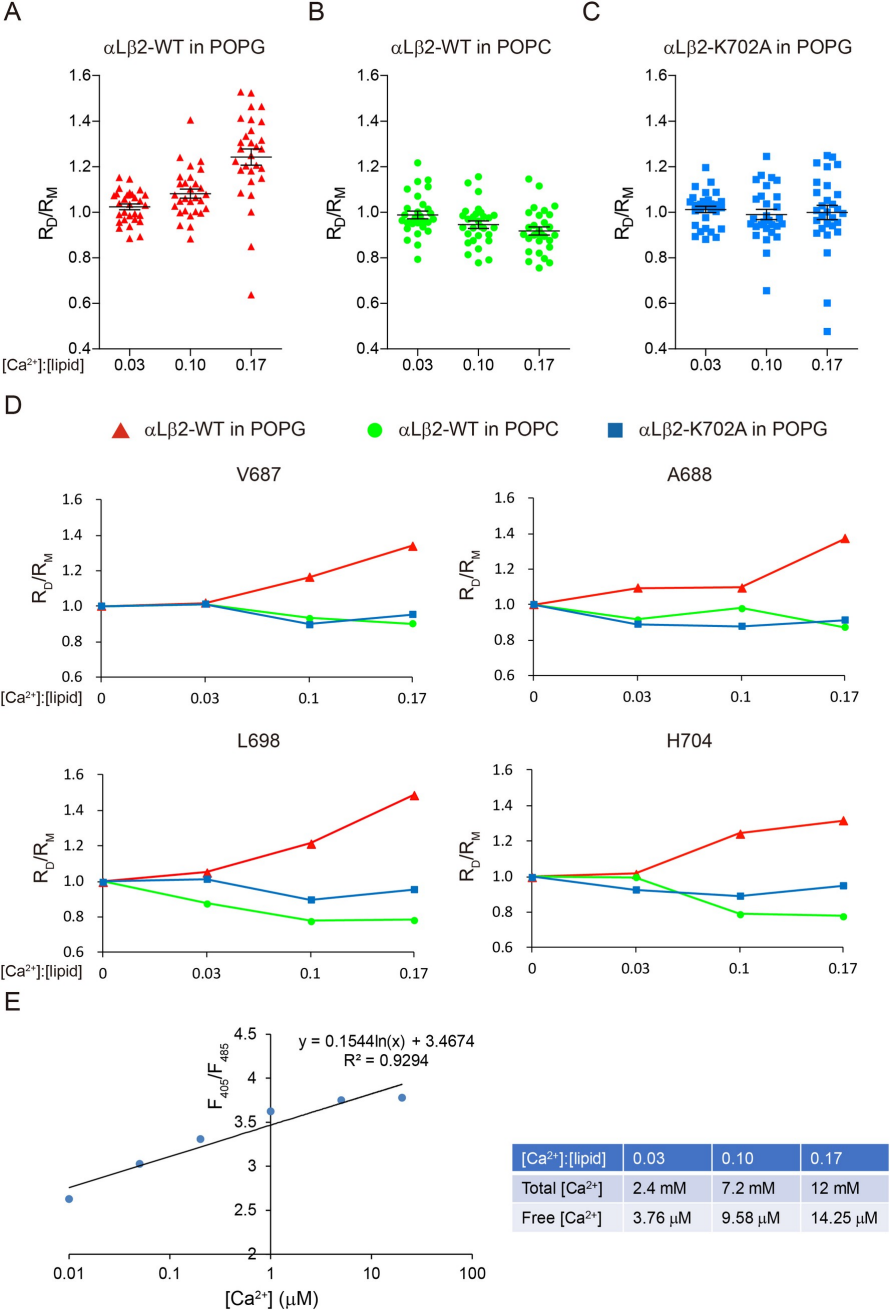
Ca^2+^ modulates intramembrane Lys–lipid interaction to destabilize αLβ2 transmembrane heterodimer. The protein samples contained 0.6 mM ^13^C/^15^N labeled β2-WT or K702A mutant, 1.2 mM unlabeled αL, 20 mM Bis-Tris (pH 6.7), 240 mM DHPC, and 72 mM POPG or POPC. Ca^2+^ was titrated into the protein sample at a ratio of Ca^2+^:phospholipid (POPC or POPG) from 0.03 to 0.17. The signal intensity changes of β2 TMD residues in response to Ca^2+^ titration in the dimer sample were normalized to that in the monomer sample. R_D_ represents I_Ca2+_/I_0Ca2+_ in the dimer sample, while R_M_ represents that ratio in the monomer sample. (A–C) Scatter plots of the R_D_/R_M_ values of αLβ2-WT TMD residues in POPG (A), POPC (B), and αLβ2-K702A TMD residues in POPG (C). Each dot represents a single β2 TMD residue. *N* = 29 for each group. Bar graphs of the R_D_/R_M_ value of each β2 TMD residue are shown in S8 Fig. (D) Line plots of the R_D_/R_M_ values of representative residues from different regions of the β2 TMD. (E) Quantification of free Ca^2+^ concentration in NMR sample. Free Ca^2+^ concentration was quantified by the 405/485 nm emission fluorescence ratio of Indo-1. The NMR sample was diluted before the measurement. The standard curve was shown on the left, and the calculated free Ca^2+^ concentration was shown on the right. The underlying data of panel A–E can be found in http://dx.doi.org/10.17632/tg2622h9dd.1. Ca^2+^, calcium ion; I_0Ca2+_, intensity under no Ca^2+^ condition; I_Ca2+_, intensity under Ca^2+^ condition; TMD, transmembrane domain; Indo-1, 2-[4-(bis(carboxymethyl)amino)-3-[2-[2-(bis(carboxymethyl)amino)-5-methylphenoxy]ethoxy]phenyl]-1H-indole-6-carboxylic acid; NMR, nuclear magnetic resonance; POPC, 1-palmitoyl-2-oleoyl-glycero-3-phosphocholine; POPG, 1-palmitoyl-2-oleoyl-sn-glycero-3-phospho-(1'-rac-glycerol); WT, wild type

The NMR system was applied to test the effect of Ca^2+^ on αLβ2 transmembrane association. Titration of calcium chloride (CaCl_2_) into the NMR sample, however, led to nonspecific signal reduction, probably due to the salt effect [48], which limits the Ca^2+^:phospholipid ratio up to 0.17 in our experiments. To dissect the specific effect of Ca^2+^ on αLβ2 dimerization, we performed Ca^2+^ titration on both αLβ2 dimer and β2 monomer samples. The signal intensity change induced by Ca^2+^ titration in the dimer sample was normalized to that in the monomer sample to filter out the interference of the nonspecific signal reduction effect. We found that Ca^2+^ increased β2 TMD signal intensity, i.e., dimer destabilization, only when acidic phospholipids were present in the membrane (Fig 6A, 6B and 6D; S8 Fig). Furthermore, when the K702 was mutated to Ala, Ca^2+^ lost its effect on destabilizing the αLβ2 dimer in the acidic phospholipid environment (Fig 6C and 6D; S8 Fig). The total Ca^2+^ concentration ranged from 2.4 to 12 mM in our experiments, but most of the Ca^2+^ cations bound to lipids so that the free Ca^2+^ concentration ranged from 3.76 μM to 14.25 μM (Fig 6E), which is within the physiological range of Ca^2+^ concentration in activated T cells. These results together show that Ca^2+^ can specifically disrupt the ionic Lys–lipid interaction to destabilize the αLβ2 transmembrane heterodimer.

### Intracellular Ca^2+^ activates αLβ2 in a signaling-independent manner

**Fig 7:**
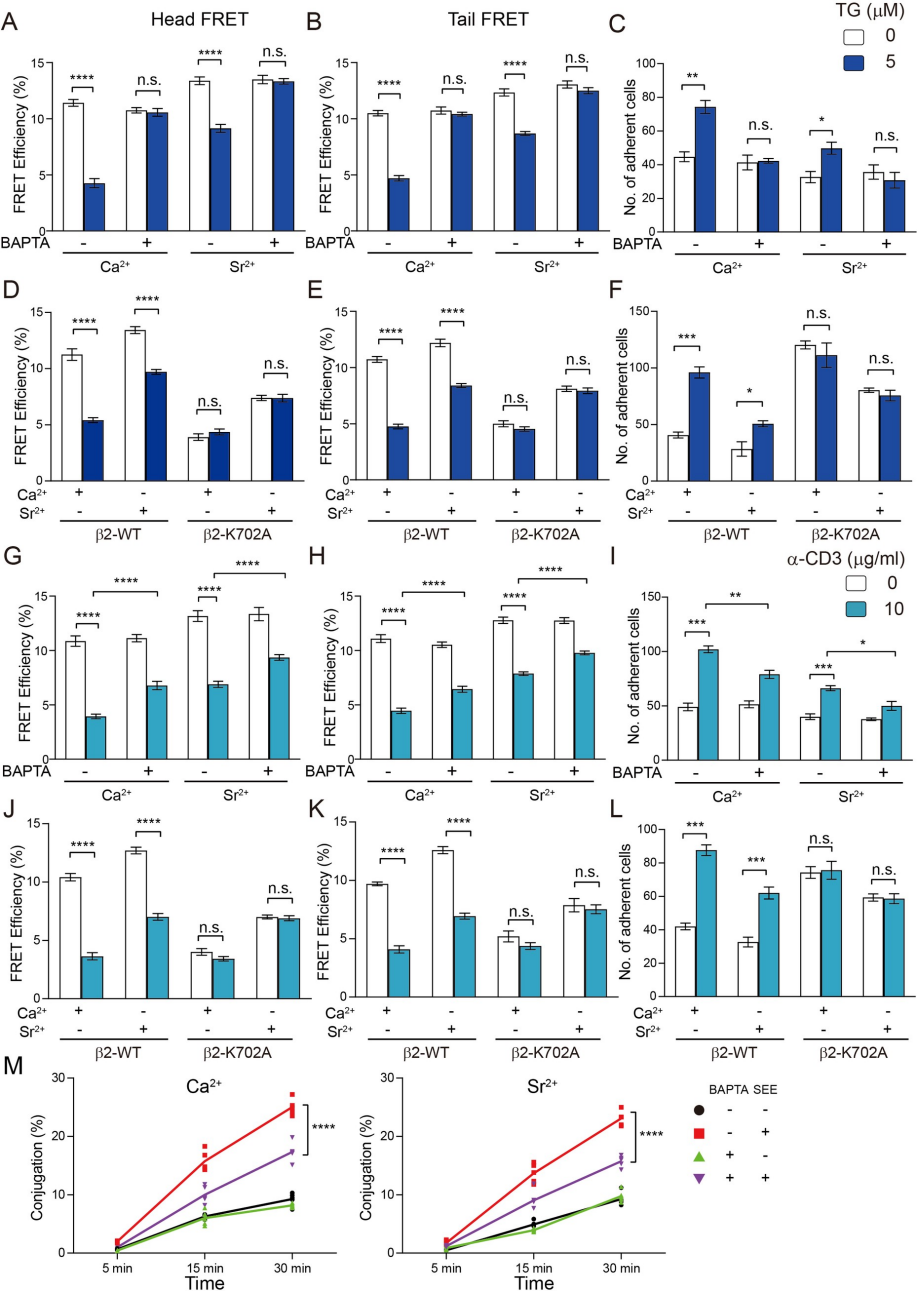
Intracellular Ca^2+^ activates αLβ2 by its charge. TG (5 μM TG in A–F) or TCR stimulation (10 μg/ml α-CD3ε in G–L) was used to induce Ca^2+^ or Sr^2+^ influx in Jurkat T cells. Pretreatment of 20 μM BAPTA-AM (A–C, G–I, M) was used to chelate intracellular Ca^2+^ or Sr^2+^. The stimulating buffer contained Ca^2+^ at physiological concentration (1 mM) or Sr^2+^ (5 mM). We used a higher Sr^2+^ concentration because Sr^2+^ influx is less efficient than Ca^2+^ influx. β2-KO Jurkat cells were reconstituted with β2-WT or K702A mutant (D–F, J–L). (A, B, D, E) WT or K702A mutant αLβ2 conformational changes induced by TG stimulation were measured by the Head and Tail FRET assays. (C, F) The adhesion of Jurkat T cells to ICAM-1-coated surface induced by TG stimulation was measured by the flow chamber assay. (G, H, J, K) WT or K702A mutant αLβ2 conformational changes induced by TCR stimulation were measured by the Head and Tail FRET assays. (I, L) The adhesion of Jurkat T cells to ICAM-1 coated surface induced by TCR stimulation was measured by the flow chamber assay. (M) Conjugation between T cells and SEE pulsed B cells measured by flow cytometry. Jurkat T cells and Raji B cells were labeled with Cell Tracker Deep Red and Cell Tracker CSFE, respectively. Raji B cells were pretreated with 5 μg/ml SEE in FBS-free RMPI-1640 medium and then mixed with Jurkat T cells in HBSS containing either 1.26 mM CaCl_2_ or 5 mM SrCl_2_. Two-way ANOVA was used to compare the differences between the group with or without BAPTA treatment in different time points (*n* = 5 for each group). Data are representatives of three independent experiments and displayed as individual points. The underlying data of panel A–M can be found in http://dx.doi.org/10.17632/tg2622h9dd.1. (A–L) Data are representatives of two independent experiments and displayed as mean ± SEM. Student *t* test was used to analyze the differences between two groups. ****P < 0.0001, ***P < 0.001, **P < 0.01. BAPTA-AM, 1,2-Bis(2-aminophenoxy)ethane-N,N,N’,N’-tetraacetic acid tetrakis (acetoxymethyl ester); Ca^2+^, calcium ion; CaCl_2_, calcium chloride; CD, cytoplasmic domain; CFSE, 5-(and-6)-Carboxyfluorescein Diacetate, Succinimidyl Ester; FBS, fetal bovine serum; FRET, florescence resonance energy transfer; HBSS, Hank’s Balanced Salt Solution; ICAM-1, intercellular adhesion molecule 1; n.s., not significant; RMPI, Roswell Park Memorial Institute; SEE, *Staphylococcus aureus* Enterotoxin E; Sr^2+^, strontium ion; SrCl_2_, strontium chloride; TCR, T-cell receptor; TG, thapsigargin; WT, wild type

We next studied the physiological role of Ca^2+^ in regulating αLβ2 function. Ca^2+^ influx induced by thapsigargin (TG) treatment led to αLβ2 activation, confirming that intracellular [Ca^2+^] elevation alone is sufficient to activate αLβ2 in T cells (Fig 7A–7C). Intracellular Ca^2+^ ions are known to have both charge-mediated function [2] and signaling-mediated function [49]. To rule out the involvement of signaling-mediated function, we replaced Ca^2+^ with strontium ion (Sr^2+^), a nonphysiological divalent cation that preserves the charge property of Ca^2+^ but loses the signaling capability in T cells [7]. Similar to Ca^2+^, Sr^2+^ induced the high-affinity conformation of αLβ2 and enhanced its adhesion to ICAM-1 (Fig 7A–7C), while it had no effect on adhesion and degranulation-promoting adaptor protein (ADAP) membrane recruitment (S9A Fig). We further examined whether integrin inside-out signaling was involved in the Ca^2+^-induced αLβ2 activation. Since the inside-out activation of integrin depends on the recruitment of adaptor proteins such as talins and kindlins to the β subunit tail [16, 18, 50, 51], we generated a cytoplasmic domain truncation mutant of β2 (β2-ΔCT) to abolish integrin inside-out signaling. Ca^2+^ or Sr^2+^ still evidently activated the tailless mutant (S9B and S9C Fig). In contrast, both Ca^2+^ and Sr^2+^ showed moderate effects in αLβ2 when the membrane-snorkeling K702 was mutated to Ala (Fig 7D–7F).

We then used T-cell receptor (TCR) crosslinking to induce Ca^2+^ influx in a more physiological way and observed evident conformational change and activation of αLβ2. The β2-ΔCT mutant had impaired adhesion, but its conformational change and ligand binding could still be enhanced by Ca^2+^ (S9D–S9G Fig). When cells were pretreated with the Ca^2+^ chelator 1,2-Bis(2-aminophenoxy)ethane-N,N,N’,N’-tetraacetic acid tetrakis (acetoxymethyl ester) (BAPTA-AM), TCR-induced αLβ2 conformational change and activation were significantly impaired (Fig 7G–7I). The β2-K702A mutant was insensitive to Ca^2+^ (Fig 7J–7L), which echoes the NMR finding in Fig 6.

We also studied the role of Ca^2+^ in T-cell conjugation with antigen presenting cells (APCs). Chelation of intracellular Ca^2+^ or Sr^2+^ diminished T-APC conjugation, showing the importance of the charge-mediated function of Ca^2+^ in regulating this process (Fig 7M).

We therefore conclude that the conformational modulation of αLβ2 by Ca^2+^ observed in the NMR experiments is physiologically relevant and critical for T-cell adhesion. Additionally, this mechanism is through the modulation of the ionic Lys–lipid interaction but not through the canonical Ca^2+^ signaling or integrin inside-out signaling.

## Discussion

Through bioinformatics analysis, we find that the presence of the membrane-snorkeling basic residue is a common feature of transmembrane proteins (Fig 1). Our NMR, MD simulations, and cell biology experiments together show that the membrane-snorkeling basic residue in integrin β2 chain has a gatekeeper function in integrin αLβ2 activity.

Mutating the K702 residue to Ala led to destabilization of αLβ2 transmembrane association in the NMR experiments (Fig 3E). This effect was dependent on the presence of acidic phospholipids in the membrane, thus suggesting that K702 might ionically interact with acidic phospholipids to regulate αLβ2 dimerization. In the studies of the platelet integrin αIIbβ3, the membrane-snorkeling Lys residue mutation in β3 chain was proposed to regulate TMD topology based on a PRE experiment [13]. This mechanism, however, has been argued by another PRE study, and therefore, this issue is left unsettled [14].

To study the mechanistic role of the membrane-snorkeling Lys in αLβ2 dimerization, we applied all-atom MD simulations because the αLβ2 dimer structure is too dynamic to be solved by NMR. The β2-K702 local region had substantial hydrophobic stacking and ionic interactions with αL. The K702A mutation caused incompact C-terminal contacts and therefore destabilized the dimer. The overall β2 transmembrane topology was not affected obviously by the mutation, which fits with the NMR observation (Fig 2G). Furthermore, we found that β2-K702, acidic phospholipid, and αL-R1094 formed ternary interactions (Fig 4D). The phosphate group of POPS could simultaneously interact with the amino group of β2-K702 and the guanidino group of αL-R1094 to stabilize the transmembrane dimer (S6 Fig). Of note, in our study, we only included several juxtamembrane but not all residues from the cytoplasmic domains of αL and β2. A previous study has reported the structure of the αLβ2 cytoplasmic domains under a lipid-free condition. The αL cytoplasmic domain was characterized by three helical segments, and extensive interactions were found between helix 1 and helix 3 of αL with β2 N-terminal cytoplasmic domain [52]. These data highlight the importance of cytoplasmic interactions in αLβ2 dimerization, and it will be interesting to further investigate this aspect under membrane condition.

Consistent with the NMR and MD simulations results, the live-cell Tail and Head FRET assays showed that the K702A mutant had further TMD/CD separation and ED extension. In contrast, the K702R mutant that preserves the positive charge showed similar conformation with WT (Fig 5B). Furthermore, the K702A mutant exhibited higher adhesion activity than WT and the K702R mutant (Fig 5C).

The above evidences showed the importance of intramembrane ionic protein–lipid interaction in regulating αLβ2 conformation and activity. Apparently, the next question was how this type of intramembrane ionic protein–lipid interaction is regulated during T-cell activation. We found that intracellular Ca^2+^ could directly use its charge to disrupt intramembrane ionic protein–lipid interaction (Figs 6 and 7), which agrees with the previous studies showing the strong binding between Ca^2+^ and the phosphate group of acidic phospholipids [7, 46, 47]. In activated T cells, Ca^2+^ influx starts in several seconds and lasts a few hours to regulate cell adhesion, activation, and differentiation [41]. Ligand engagement of TCRs or chemokine receptors can lead to Ca^2+^ influx in T cells. One major effect of Ca^2+^ influx is to induce a stop signal to sustain stable T-cell contact with antigen presenting cells or target cells and the formation of immunological synapse [43]. Inhibition of CRAC channel, the major Ca^2+^ channel in T cells, with a dominant-negative Orai1 mutant (E106A) significantly impaired T-cell basal mobility and chemokine-induced homing [45, 53]. Earlier studies suggested that Ca^2+^ could induce talin cleavage to activate αLβ2 but this mechanism remains to be further clarified because another study reported that talin cleavage could not be detected after Ca^2+^ influx [54–56]. Our experiments showed that intracellular Ca^2+^ could directly disrupt intramembrane Lys–lipid interaction to activate αLβ2. The NMR experiments showed that Ca^2+^ could destabilize αLβ2 transmembrane association only when acidic phospholipids were present in the membrane and the conserved Lys residue was intact (Fig 6 and S8 Fig). Separation of αLβ2 transmembrane domain could cause an allosteric effect on the extracellular domain to induce high-affinity conformation [26, 57, 58]. Indeed, FRET measurements showed that intracellular Ca^2+^ induced the high-affinity conformation of αLβ2-WT and enhanced its adhesion with ICAM-1 (Fig 7A–7C). Such an effect was not observed for the αLβ2-K702A mutant. Given the fact that Ca^2+^ is an important second messenger that regulates various signaling pathways, we used Sr^2+^ to replace Ca^2+^ to eliminate its signaling function but preserve the charges. Sr^2+^ could still trigger αLβ2 activation without activation of the canonical LAT-SLP76-ADAP pathway (Fig 7D–7F and S9 Fig), supporting the notion that Ca^2+^ can modulate αLβ2 conformation and activity via its charge property. Nevertheless, we did notice that the effect of Sr^2+^ on αLβ2 was less robust than Ca^2+^, suggesting that Ca^2+^ signaling could also regulate αLβ2 activation. The canonical inside-out signaling pathway of integrin was not required for the charge effect of Ca^2+^ because the β2 cytoplasmic domain truncation mutant could be still activated by Ca^2+^ or Sr^2+^.

In conclusion, our study unveils a new charge-based regulation of αLβ2 activity that might have general relevance to other membrane proteins that contain membrane-snorkeling basic residues (S10 Fig). It provides a mechanistic explanation for the well-recognized stop signal caused by intracellular Ca^2+^ in T cells. Notably, it has been already shown that Ca^2+^ can amplify TCR and CD28 signaling [5, 7] by interfering juxtamembrane protein–lipid interaction, and now, we show Ca^2+^ can further activate the major integrin molecule in T cells by interfering with intramembrane protein–lipid interaction. It is known that tumor-infiltrating T cells have defect in αLβ2 activation, which causes cytokine secretion problem [59]. Ca^2+^ can be considered as a potential target for boosting T-cell antitumor immunity through the modulations of T-cell adhesion and other aspects such as signaling and metabolism [60–62].

## Materials and methods

### Protein expression and purification

The human integrin β2 TMD construct (V667 to Y713) was expressed as a TrpLE fusion protein in *Escherichia coli* BL21 (DE3) cells. C673S substitution was incorporated in the β2 construct to avoid peptide cross-linking and aggregation, and a His_9_ tag was added to the N-terminus for purification purposes. The His_9_-TrpLE-β2 fusion protein was expressed in inclusion body after IPTG induction and dissolved in a denaturing buffer that contains 50 mM Tris-HCl (pH 8.0), 6 M Guanidine hydrochloride, 200 mM NaCl, and 1% Triton X-100. The fusion protein was then subjected to affinity purification using a Ni-NTA affinity column (Genescript). Purified peptide was cleaved at the Asp-Pro site between TrpLE and β2 in 10% formic acid containing 6 M Guanidine-HCl. The digest was dialyzed to water, lyophilized, and dissolved in 50% TFA before HPLC purification by a ZORBAX 300SB-C3 column. The elution was performed with a linear gradient of 20%–80% Buffer B (acetonitrile, 0.2% TFA) in 60 min. The purity of β2 TMD peptide was assessed by SDS-PAGE and mass spectrometry. The αL TMD peptide (K1055-K1099) was chemically synthesized by Neobioscience company.

### Bioinformatics analysis of membrane-snorkeling basic residues in transmembrane proteins

Predicted transmembrane domains of single-span transmembrane proteins in the Membranome database [28] were checked manually to ensure both ends of the transmembrane domain are hydrophobic residues. Single-span transmembrane proteins from yeast (*S*. *cerevisiae)* and human (*H*. *sapiens)* were selected for further analysis. We also selected the human single-span transmembrane proteins localized at the plasma membrane as an independent data set because the plasma membrane contains the highest amount of acidic phospholipids in cell membrane systems. The percentages of single-spanning transmembrane proteins containing Lys only, Arg only, both Lys and Arg, or no Lys or Arg were analyzed. Moreover, location of the basic residue in TMD was analyzed for proteins with known transmembrane topology.

### NMR sample preparation

Lipid bicelles (Q = 0.3) with different lipid compositions were prepared as previously reported [4]. The bicelle solution was applied to dissolve lyophilized αL and β2 peptide and multiple rounds of freezing-thawing were performed to enable successful protein reconstitution into the membrane bilayer. Bis-Tris (pH 6.7) stock buffer solution was added into the sample to make the final concentration 20 mM. The NMR sample of β2 monomer contained 0.6 mM ^13^C/^15^N-labeled β2 TMD peptide, 20 mM Bis-Tris (pH 6.7), 240 mM DHPC, 72 mM phospholipids (100% POPC, 100% POPG, or 67% POPC/ 33% POPG), 1 × protease inhibitor cocktail (Thermo Fisher), and 0.2% NaN_3_. In the αLβ2 transmembrane heterodimer sample, an additional 1.2 mM αL peptide was reconstituted into the bicelles. All NMR samples contained 10% D_2_O (v/v) for frequency lock.

### NMR spectroscopy

NMR experiments were conducted at 30°C on Agilent ASC 600 MHz, Bruker AVANCE III 600, and 900 MHz spectrometers equipped with cryogenic probes. Sequence-specific assignment of the backbone chemical shifts was accomplished using triple resonance experiments, including HNCA, HNCACB, CBCA(CO)NH, HNCO, and ^15^N-edited NOESY-HSQC with a mixing time of 150 ms. β2-K702A assignments were transferred from the wild type using HNCA experiment.

For the Ca^2+^ titration experiments, Ca^2+^ dissolved in 240 mM DHPC solution was added to the sample to reach the [Ca^2+^]:[phospholipid] molar ratio from 0.03–0.17 (the absolute [Ca^2+^] ranged from 2.4 mM to 12 mM). Higher Ca^2+^ concentration caused sample precipitation.

Hydrogen-deuterium (H/D) exchange experiments were performed to obtain hydrogen bond information for integrin β2 TMD structure calculation. A series of time-dependent ^15^N-TROSY spectra were measured after D_2_O was added into the lyophilized β2 TMD NMR sample, and the peak intensity for each residue was traced. The hydrogen bond restraints were applied to the slowly disappeared residues.

The signal intensity of each residue in the ^15^N-TROSY spectra was analyzed by NMRView [63] and KUJIRA [64]. Chemical shift perturbation (CSP) was calculated by the formula Δδ (HN/^15^N) = ((ΔδHN)^2^ + (0.154* Δδ^15^N)^2^)^1/2^.

### Structure calculation

NOE distance restrains to calculate the structure of β2 TMD were obtained from ^15^N-edited NOESY, ^13^C-edited aliphatic, and aromatic NOESY with 150 ms mixing times. Backbone dihedral angle restraints (ϕ and ψ) were derived from ^13^CO, ^13^C^α^, ^13^C^β^, ^1^H^α^, and ^15^N^H^ chemical shift values using TALOS+ [65]. The short range and medium range NOE connectivities were used to establish the sequence-specific ^1^H NMR assignment and to identify elements of the regular secondary structure. Hydrogen bond restraints were added according to the H/D exchange experiment. Structure calculations were performed using CYANA 2.1 and visualized using MOLMOL and PyMOL. A total of 100 structures were calculated, and the 20 structures with the lowest target function values were selected. The statistics of the structures as well as the restraints used for the structure calculation are summarized in S1 Table.

### Ca^2+^ quantification

To quantify the Ca^2+^ concentration in NMR samples, bicelle with different concentrations of Ca^2+^ was first diluted, and the lipid was extracted by chloroform. The supernatant was collected and Indo-1 was then added to the sample and standard Ca^2+^ solution. The florescence was measured by Bio Tek SynergyNEO. F405/F485 was used to quantify Ca^2+^ concentration.

### Generation of Jurkat T cell line expressing mutant β2 subunit

CRISPR-Cas9 mediated gene editing was used to knockout the endogenous β2 in Jurkat T cells. The sgRNA sequence targeting β2 subunit was 5′-CCGGGAATGCATCGAGTCGGGGC-3′. Jurkat T cells were transfected with the recombinant lentivirus expressing β2 sgRNA to generate a stable β2 knockout (β2-KO) cell line. Then, sgRNA-resistant β2-WT, β2-K702A, β2-K702R, or β2-ΔCT mutants were reconstituted in β2-KO Jurkat T cells. To determine the surface expression of reconstituted β2 on β2-KO Jurkat T cells, the cells were stained with α-β2 (TS1/18) and analyzed by flow cytometry.

For the Tail FRET measurement, αL-mTurquoise2 and β2-mCitrine were expressed in αL/β2 double KO Jurkat T cells. The sgRNA sequence targeting αL subunit was 5′-GCCGGCCTCGAGCTACAACC-3′.

### Modulation of intracellular Ca^2+^ concentration

To induce Ca^2+^ influx, Jurkat T cells were stimulated with 5 μM TG or 10 μg/ml α-CD3ε (UCHT-1) at 37°C in the stimulation buffer (HBS buffer containing 1 mM Ca^2+^) for 5 min. To rule out the role of Ca^2+^ signaling in αLβ2 activation, we replaced Ca^2+^ by Sr^2+^ in the stimulation buffer. Sr^2+^ preserves the charge property of Ca^2+^ but cannot trigger Ca^2+^ signaling pathways [7]. Sr^2+^ can influx via the same CRAC channel as Ca^2+^ while its conductivity is lower [66]. Higher Sr^2+^ concentration (5 mM) was therefore used in the stimulation buffer to achieve similar influx level as Ca^2+^.

To chelate intracellular Ca^2+^, Jurkat T cells were pretreated with 20 μM BAPTA-AM in 37°C for 30 min, followed by two rounds of washing.

### FRET measurement and analysis

In the extracellular Head FRET assay, Jurkat T cells were washed twice with HBSS containing 5 mM EDTA and then resuspended with HBSS. Cells were then seeded on poly-L-lysine (100 μg/ml) substrates and incubated for 10 min at 37°C. For TG or α-CD3ε (UCHT-1) treatment, cells were pretreated with 5 μM TG or 10 μg/ml α-CD3ε (UCHT-1) at 37°C under different cation conditions for 5 min. Then, cells were fixed with 3.7% paraformaldehyde (PFA)/HBSS for 20 min at room temperature, and nonspecific sites were blocked by HBSS containing 2% BSA at room temperature for 30 min. Then, cells were stained with 20 μg/ml TS2/4 Fab conjugated with Alexa488 for 30 min at 37°C. After two rounds of washing, cells were labeled with 10 μM FM-4-64 FX for 4 min on ice, washed once, fixed, and mounted with Mowoil under a coverslip.

In the intracellular Tail FRET assay, Jurkat T cells expressing αL-mTurquoise2/β2-mCitrine were treated as above. Then, cells were fixed with 3.7% PFA in HBSS for 20 min at room temperature and subjected to photobleaching FRET imaging.

FRET image acquisition, image registration, background subtraction, and data analyses were performed with Leica TCS SP8 under a 63 × oil objective. FRET efficiency E was calculated as E = 1 –(Fdonor(d)_Pre_/Fdonor(d)_Post_), in which Fdonor(d)_Pre_ and Fdonor(d)_Post_ are the mean donor emission intensities of mTurquoise2 pre- and post-photobleaching. Cells with comparable labeling (Head FRET) or comparable αL-mTurquoise2/β2-mCitrine expression levels (Tail FRET) were used for FRET data quantitation.

### Flow chamber assay

A polystyrene Petri dish was coated with a 5 mm-diameter, 20 μl spot of 20 μg/ml purified h-ICAM-1/Fc in coating buffer (PBS, 10 mM NaHCO_3_ [pH 9.0]) for 1 h at 37°C, followed by 2% BSA in coating buffer for 1 h at 37°C to block nonspecific binding sites. Cells were washed twice with HBSS containing 5 mM EDTA and 0.5% BSA before resuspension at 1 × 10^7^/ml density in buffer A (HBSS containing 0.5% BSA). Cells were then diluted to 1 × 10^6^/ml in buffer A containing different divalent cations immediately before infusion into the flow chamber using a Harvard apparatus programmable syringe pump.

Cells were allowed to accumulate for 30 s at 0.3 dyne/cm^2^ and for 10 s at 0.4 dyne/cm^2^. Then, shear stress was increased every 10 s from 1 dyne/cm^2^ up to 32 dynes/cm^2^ in 2-fold increments. The number of bound cells remained at the end of each 10-s interval was determined.

### T cell–target cell conjugation assay

Jurkat T cells expressing β2-WT or β2-K702A and Raji B cells were labeled with Cell Tracker CFSE and Cell Tracker Deep Red, respectively. For antibody blocking, Jurkat T cells were incubated with 10 μg/ml α-CD2 (clone: RPA-2.10) or α-LFA-1 (clone: TS1/18) for 15 min at 37°C. To chelate intracellular Ca^2+^, Jurkat T cells were pretreated with 20 μM BAPTA-AM in 37°C for 30 min. For *Staphylococcus aureus* Enterotoxin E (SEE) stimulation, Raji B cells were pretreated with 5 μg/ml SEE in FBS-free RPMI-1640 medium for 30 min at 37°C. After washing, Jurkat T cells and Raji B cells were resuspended in RPMI-1640 medium or HBSS at 1 × 10^6^ cells/ml. They were mixed at the 1:1 ratio and allowed to interact for 5–30 min at 37°C. Cells were then resuspended, fixed, and analyzed by flow cytometry. The percentage of conjugation was calculated as the percentage of dual-labeled (red/green) events.

### Soluble ICAM-1–binding assay

Cells were washed with Hank’s Balanced Salt Solution (HBSS) containing 5 mM EDTA and then resuspended in HBSS containing either 1 mM CaCl_2_ and 1 mM MgCl_2_ or 5 mM SrCl_2_ and 1 mM MgCl_2_. Cells were pretreated with 10 μg/ml anti-CD3ε mAb (clone: UCHT-1) or an equal volume of HBSS for 5 min at 37°C, followed by an incubation with 20 μg/ml ICAM-1-Fc and Alexa Fluor 647-conjugated anti-human-IgG antibody (Invitrogen) for 20 min at 37°C. Cells were fixed in 3.7% PFA for 10 min and then washed in HBSS. ICAM-1 binding was evaluated by flow cytometry.

### Isolation of plasma membrane fractions

Jurkat T WT or LAT-KO cells (1 × 10^7^) were either left untreated or stimulated for 5 min with 10 μg/ml anti-CD3ε mAb (clone: UCHT-1). Then, cells were washed with PBS and resuspended on ice in a hypotonic buffer. Cells were sheared, and nuclei and unbroken cells were removed by low-speed centrifugation. The remaining supernatant was recentrifuged, and the cytosolic fraction (supernatant) was collected. The remaining pellet (membrane fraction) was washed twice with hypotonic buffer and finally resuspended on ice in lysis buffer. Protein concentrations of the cytosolic and membrane fractions were quantified by BCA assay and active Rap1 was isolated using a glutathione GST-RalGDS-Rap1 binding domain (RBD) fusion protein. Equivalent masses of cytosolic and membrane fractions were loaded onto 10% or 12% SDS-PAGE gels for separation.

### Molecular dynamics simulations

The monomer of αL (PDB code 2M3E) and β2 (PDB code 5ZAZ) were used to construct the dimer of αLβ2, which is based on the dimer conformation of αIIbβ3 (PDB code 2K9J) [67]. The assembling of αLβ2-WT and αLβ2-K702A mutant into the bilayer was employed using the CHARMM-GUI web server [68]. To mimic the charge distribution in plasma membrane, an asymmetric model membrane consisted of total 120 POPS/POPC lipid molecules, with 100% POPC lipids in the outer leaflet and POPS/POPC lipids with a mixture ratio of 1:2 in inner leaflet was applied. Then the systems were solvated with TIP3P water molecules and the charges of the systems were balanced to neutral using 150 mM NaCl. All MD simulations were first performed using AMBER16 package [69] with CHARMM36 force field [70] under NPT condition for 380 ns, and the representative conformation of the largest cluster was then chosen as the initial structure to perform new MD simulation based on the polarizable atomic multipole-based force field. Here, the parametrization for the lipid adopts the same protocol as our previous work [38, 71]. The simulations were performed under the NPT ensemble at 303.5 K and 1 bar with an Andersen Thermostat [72] and a Monte Carlo anisotropic barostat implemented in the OpenMM package [73]. The Rattle algorithm [74] was also adopted in the MD to constrain all bonds involving the hydrogen, which ensured the stability of the system with a 4 fs integration time step. The Particle-Mesh Ewald (PME) [75, 76] method for long range electrostatic calculations was employed, and the cutoff was set to 10.0 Å. The cutoff for the nonbonded van der Waals interactions was set to 12.0 Å. Mutual polarization was used, which employed the self-consistent field (SCF) with the Direct Inversion in Iterative Subspace (DIIS) method [77] to calculate the induced dipole moment in every integration. The convergence threshold of the induced dipole in SCF iteration was set to 0.00001D. The last 50 ns of each simulation were used for analysis, and the analysis of interaction energies, native contacts, etc. were performed by cpptraj module [78] in AMBER16 [69].

Spatial distribution function (SDF), which reflects the average 3D density distribution, has been applied to investigate the distribution of POPS around the αLβ2-WT and αLβ2-K702A. In each condition, the SDF is calculated based on the snapshot configurations of the last 50 ns in 10 independent MD trajectories. The space is divided by the voxel element with [1 * 1 * 1] Å^3^. And the number of POPS atoms in each grid in the inner leaflet ([−40, 40]*[−40, 40]*[−40, 0] Å^3^) is counted. Therefore, the peaks of SDF imply the locations where POPS molecules reside with high probability. The spatial distribution of POPS around αLβ2-WT and αLβ2-K702A in the inner leaflet is projected onto the membrane (xy) plane.

### Statistical information

All statistical analyses were performed using GraphPad Prism 7 software. Student *t* test was used to compare the FRET efficiencies and numbers of adherent cells. Paired *t* test was used to compare the dimer formation in different lipids. Two-way ANOVA was performed to compare the difference between groups. Statistical methods and significance values were stated in the figure legends. The repeats were independent cells or protein samples.

## Supporting information

S1 Table NMR and refinement statistics for integrin β2 TMD monomer. (DOCX)

S1 Fig K702 is embedded in the lipid bicelle. Peak intensity changes induced by the addition of 5 mM 16-DSA on integrin β2 TMD. *I/I*_*0*_ of ^15^N TROSY was used to quantify the paramagnetic effect. *I* represents the signal intensity with 16-DSA, while *I*_0_ represents the signal intensity without 16-DSA. The underlying data can be found in http://dx.doi.org/10.17632/tg2622h9dd.1. ^15^N, nitrogen-15; 16-DSA, 16-doxyl stearic acid; *I/I*_*0*_, intensity ratio; TMD, transmembrane domain.
(TIF)

S2 Fig Intensity changes of β2 TMD upon unlabeled αL and β2 titration. ^15^N-labeled β2-WT was mixed with different concentration of unlabeled αL (A) or β2 (B) in the mixture lipid bicelles. The β2-WT monomer contained 0.6 mM ^15^N-labeled β2 TMD peptide, 20 mM Bis-Tris (pH 6.7), 240 mM DHPC, 24 mM POPG, and 48 mM POPC. Superimposed ^1^H-^15^N TROSY-HSQC spectra of ^15^N-labeled β2-WT in the presence or absence of different concentrations of unlabeled αL (A) or β2 (B) are shown on left. Signal intensity comparisons of β2 TMD residues are shown on the right. *I* represents the signal intensity of β2 TMD residues with unlabeled αL or β2, while *I*_0_ represents the corresponding one without unlabeled αL or β2. The underlying data can be found in http://dx.doi.org/10.17632/tg2622h9dd.1. ^1^H, hydrogen-1, ^15^N, nitrogen-15; HSQC, heteronuclear single quantum coherence; POPC, 1-palmitoyl-2-oleoyl-glycero-3-phosphocholine; POPG, 1-palmitoyl-2-oleoyl-sn-glycero-3-phospho-(1'-rac-glycerol); TMD, transmembrane domain; TROSY, transverse relaxation-optimized spectroscopy; WT, wild type.
(TIF)

S3 Fig αLβ2 heterodimer formation in different lipid conditions. ^15^N-labeled β2-WT was mixed with unlabeled αL in different concentration (total lipid concentration ranging from 36 mM to 144 mM) (A) or size (*q* value ranging from 0.3 to 0.5) (B) of mixture lipid bicelles to form heterodimer. The β2-WT monomer contained 0.6 mM ^15^N-labled β2 TMD peptide. In the αLβ2 transmembrane heterodimer sample, additional 1.2 mM αL peptide was reconstituted into the bicelles. ^15^N, nitrogen-15; TMD, transmembrane domain; WT, wild type.
(TIF)

S4 Fig Stabilization of αLβ2 transmembrane association by acidic phospholipids. ^1^H-^15^N TROSY-HSQC spectra of αLβ2 dimer reconstituted in POPC, mixture (33% POPG, 67% POPC), or POPG bicelles. β2 was labeled by ^13^C/^15^N and αL was unlabeled. The full spectra are shown in (A–C) and representative residues from extracellular domain, transmembrane domain, and cytoplasmic domain are shown in (D). Signal intensity reductions of β2 TMD residues upon dimer formation in different lipid bicelles are shown I(E). *I* represented the signal intensity of β2 TMD residue in the dimer sample, while *I*_0_ represented the corresponding one in the monomer sample. The underlying data can be found in http://dx.doi.org/10.17632/tg2622h9dd.1. ^1^H, hydrogen-1; ^15^N, nitrogen-15; HSQC, heteronuclear single quantum coherence; POPC, 1-palmitoyl-2-oleoyl-glycero-3-phosphocholine; POPG, 1-palmitoyl-2-oleoyl-sn-glycero-3-phospho-(1'-rac-glycerol); TMD, transmembrane domain; TROSY, transverse relaxation-optimized spectroscopy.
(TIF)

S5 Fig Minor effect of acidic phospholipids on β2 monomer. (A) ^1^H-^15^N TROSY-HSQC spectra of β2 monomer reconstituted in different lipid bicelles, i.e., POPC mixture (33% POPG, 67% POPC) or POPG bicelles. (B) Peak intensity of β2 residues in different lipid bicelles. The underlying data can be found in http://dx.doi.org/10.17632/tg2622h9dd.1. ^1^H, hydrogen-1; ^15^N, nitrogen-15; HSQC, heteronuclear single quantum coherence; POPC, 1-palmitoyl-2-oleoyl-glycero-3-phosphocholine; POPG, 1-palmitoyl-2-oleoyl-sn-glycero-3-phospho-(1'-rac-glycerol); TMD, transmembrane domain; TROSY, transverse relaxation-optimized spectroscopy.
(TIF)

S6 Fig Interaction energy of pairwise atoms between POPS and β2-K702/αL-R1094. The Y-axis indicates the atom index of POPS. The x-axis indicates the atom index of αL-R1094 (left) or β2-K702 (right). The values of the interaction energy, in the range of [−20, 20], are shown as blue (negative) and red (positive) according to the color bar. The interaction energy of first 35 atoms of POPS with αL-R1094 or β2-K702 are enlarged and shown in the middle. The interaction pairs with the lowest interaction energy are highlighted by rounded rectangle in red color. POPS, 1-palmitoyl-2-oleoyl-sn-glycero-3-phospho-L-serine.
(TIF)

S7 Fig The role of CD2 in T-APC conjugation. T-cell adhesion to target cells was measured by flow cytometry. Jurkat T cells and Raji B cells were labeled with Cell Tracker CSFE and Cell Tracker Deep Red, respectively. To block CD2-CD58 interaction, Jurkat T cells were pretreated with 10 μg/ml α-CD2 (RPA-2.10). Representative FACS pictures are shown at the left. The conjugates appear at the right upper corner. The underlying data can be found in http://dx.doi.org/10.17632/tg2622h9dd.1. Two-way ANOVA was used to compare the differences between β2-WT and β2-K702A in different time points (*n* = 5 for each group). Data are representative of three independent experiments and displayed as individual points. ****P < 0.0001. APC, antigen presenting cell; CD, cytoplasmic domain; CFSE, 5-(and-6)-Carboxyfluorescein Diacetate, Succinimidyl Ester; FACS, fluorescence-activated cell sorting; WT, wild type.
(TIF)

S8 Fig The effect of Ca^2+^ on αLβ2 dimerization. Peak intensity changes of each β2 TMD residue under Ca^2+^ titration are displayed as a bar graph. R_D_/R_M_ values of αLβ2-WT in POPG (A), POPC (B), and αLβ2-K702A in POPG (C) are shown. R_D_ represents I_Ca2+_/I_0Ca2+_ in the dimer sample, while R_M_ represents that ratio in the monomer sample. Ca^2+^:phospholipid (POPC or POPG) was from 0.03 to 0.17. The underlying data can be found in http://dx.doi.org/10.17632/tg2622h9dd.1. Ca^2+^, calcium ion; I_0Ca2+_, intensity under no Ca^2+^ condition; I_Ca2+_, intensity under Ca^2+^ condition; POPC, 1-palmitoyl-2-oleoyl-glycero-3-phosphocholine; POPG, 1-palmitoyl-2-oleoyl-sn-glycero-3-phospho-(1'-rac-glycerol); TMD, transmembrane domain; WT, wild type.
(TIF)

S9 Fig Tailess β2 shows impaired adhesion but can still be activated. (A) Sr^2+^ does not cause membrane recruitment of ADAP and Rap1. Western blot analysis of ADAP and GTP-Rap1 recruitment to plasma membrane in WT and LAT-KO Jurkat T cells. Cells were either left unstimulated or stimulated with 5μM TG or 10 μg/ml α-CD3ε (UCHT-1) in HBSS containing 5 mM Sr^2+^/1 mM Mg^2+^ for 5 min and subjected to cytosolic and plasma membrane fractionation. Active Rap1 (GTP-Rap1) was isolated using a GST-RalGDS-Rap1 binding domain fusion protein. To control the fractionation efficiency, fractions were assessed for the presence of CD11a and β-actin. (B–E) β2-KO Jurkat cells were reconstituted with β2-WT, cytoplasmic domain truncation mutant. WT or cytoplasmic domain truncation mutant (ΔCT) αLβ2 conformational changes induced by TG (B, C) or TCR (D, E) stimulation were measured by the Head and Tail FRET assays. (F) Adhesive modality of Jurkat T cells expressing WT or ΔCT mutant αLβ2 on ICAM-1 substrates at a wall shear stress of 0.4 dyn/cm^2^ (left panel) and 1 dyn/cm^2^ (right panel). (G) Binding of soluble ICAM-1 to Jurkat T cells expressing WT or ΔCT mutant αLβ2 treated with or without 10 μg/ml α-CD3ε (UCHT-1) in HBSS containing 1 mM Ca^2+^/ Mg^2+^ or 5 mM Sr^2+^/1 mM Mg^2+^. ICAM-1 binding was measured by flow cytometry and presented as MFI normalized to integrin expression (TS1/18 binding). The underlying data of panel B–G can be found in http://dx.doi.org/10.17632/tg2622h9dd.1. Data are representative of two independent experiments and displayed as mean ± SEM. Student *t* test was used to analyze the differences between two groups. *P < 0.05; **P < 0.01, ***P < 0.001, ****P < 0.0001. ADAP, adhesion and degranulation-promoting adaptor protein; Ca^2+^,calcium ion; CD, cytoplasmic domain; FRET, fluorescence resonance energy transfer; HBSS, Hank’s Balanced Salt Solution; ICAM-1, intercellular adhesion molecule 1; MFI, mean fluorescence intensity; Mg^2+^, magnesium ion; n.s., not significant; Sr^2+^, strontium ion; TCR, T-cell receptor; TG, thapsigargin; WT, wild type.
(TIF)

S10 Fig Ca^2+^-mediated αLβ2 activation model. (A) In resting T cells, the ionic interaction between the β2-K702 amino group and the phosphate group of acidic phospholipids stabilizes transmembrane association between αL and β2 subunits, thus keeping αLβ2 in low-affinity conformation. (B) In activated T cells, Ca^2+^ ions quickly influx and generate high local [Ca^2+^] [5, 7]. Local Ca^2+^ ions can directly neutralize the lipid phosphate group to destabilize αLβ2 transmembrane association, thus turning αLβ2 to high-affinity conformation. This effect is independent of Ca^2+^ downstream signaling and integrin inside-out signaling. Ca^2+^, calcium ion.
(TIF)

## Abbreviations

^1^H: hydrogen-1
^15^N: nitrogen-15
^13^C: carbon-13
ADAP: adhesion and degranulation-promoting adaptor protein
APC: antigen presenting cell
BAPTA-AM: 1,2-Bis(2-aminophenoxy)ethane-N,N,N’,N’-tetraacetic acid tetrakis (acetoxymethyl ester)
DHPC: 1,2-dihexanoyl-sn-glycero-3-phosphocholine
DIIS: Direct Inversion in Iterative Subspace
16-DSA: 16-doxyl stearic acid
Ca^2+^: calcium ion
CaCl_2_: calcium chloride
CD: cytoplasmic domain
CFSE: 5-(and-6)-Carboxyfluorescein Diacetate, Succinimidyl Ester
CSP: chemical shift perturbation
CRAC: calcium release–activated channels
CYANA: combined assignment and dynamics algorithm for NMR applications
ED: extracellular domain
FACS: fluorescence-activated cell sorting
FBS: fetal bovine serum
FRET: fluorescence resonance energy transfer
HBSS: Hank’s Balanced Salt Solution
H/D: hydrogen-deuterium
HSQC: heteronuclear single quantum coherence
ICAM-1: intercellular adhesion molecule 1
Indo-1: 2-[4-(bis(carboxymethyl)amino)-3-[2-[2-(bis(carboxymethyl)amino)-5-methylphenoxy]ethoxy]phenyl]-1H-indole-6-carboxylic acid
LFA-1: lymphocyte function-associated antigen 1
MD: molecular dynamics
MFI: mean fluorescence intensity
Mg^2+^: magnesium ion
NMR: nuclear magnetic resonance
PFA: paraformaldehyde
PM: plasma membrane
PME: Particle-Mesh Ewald
POPC: 1-palmitoyl-2-oleoyl-glycero-3-phosphocholine
POPG: 1-palmitoyl-2-oleoyl-sn-glycero-3-phospho-(1'-rac-glycerol)
POPS: 1-palmitoyl-2-oleoyl-sn-glycero-3-phospho-L-serine
PRE: paramagnetic relaxation enhancement
RPD: Rap1 binding domain
RPMI: Roswell Park Memorial Institute
SCF: self-consistent field
SDF: spatial distribution function
SEE: Staphylococcus aureus Enterotoxin E
Sr^2+^: strontium ion
SrCl_2_: strontium chloride
TCR: T-cell receptor
TG: thapsigargin
TMD: transmembrane domain
TROSY: transverse relaxation-optimized spectroscopy
WT: wild type
