# Supplementary material for: Intramembrane ionic protein–lipid interaction regulates integrin structure and function
Source: PLoS Biol. 2018 Nov 14;16(11):e2006525. doi: 10.1371/journal.pbio.2006525 (PMC6261646; doi:10.1371/journal.pbio.2006525)
Supplement: S1 Table — (DOCX) [file pbio.2006525.s001.docx]

**S1 Table. NMR and refinement statistics for integrin β2 TMD monomer**

|  | **Protein (PDB** 5ZAZ**)** |
| --- | --- |
| **NMR distance and dihedral constraints** |  |
| Distance constraints |  |
| Total NOE | 452 |
| Intraresidue | 161 |
| Inter-residue | 291 |
| Sequential (\|*i* – *j*\| = 1) | 178 |
| Medium range (2 ≤ \|*i* – *j*\| ≤ 4) | 113 |
| Long range (\|*i* – *j*\| ≥ 5) | 0 |
| Hydrogen bonds | 50 |
| Total dihedral-angle restraints |  |
| *φ* | 33 |
| *ψ* | 33 |
|  |  |
| **Structure statistics** |  |
| CYANA target function value  Violations (mean ± s.d.) | 0.20 |
| Distance constraints (Å) | 0 |
| Dihedral-angle constraints (°) | 0 |
| Max. dihedral-angle violation (°) |  |
| Max. distance-constraint violation (Å) |  |
| Average pairwise r.m.s. deviation (Å)^a^ |  |
| Heavy | 0.66 |
| Backbone | 0.31 |

^a^Pairwise r.m.s.d. was calculated for the α-helix region (I679-E712) for the final 20 structures with the lowest target function values.
