# Supplementary figures and images for: Intramembrane ionic protein–lipid interaction regulates integrin structure and function
Source: PLoS Biol. 2018 Nov 14;16(11):e2006525. doi: 10.1371/journal.pbio.2006525 (PMC6261646; doi:10.1371/journal.pbio.2006525)

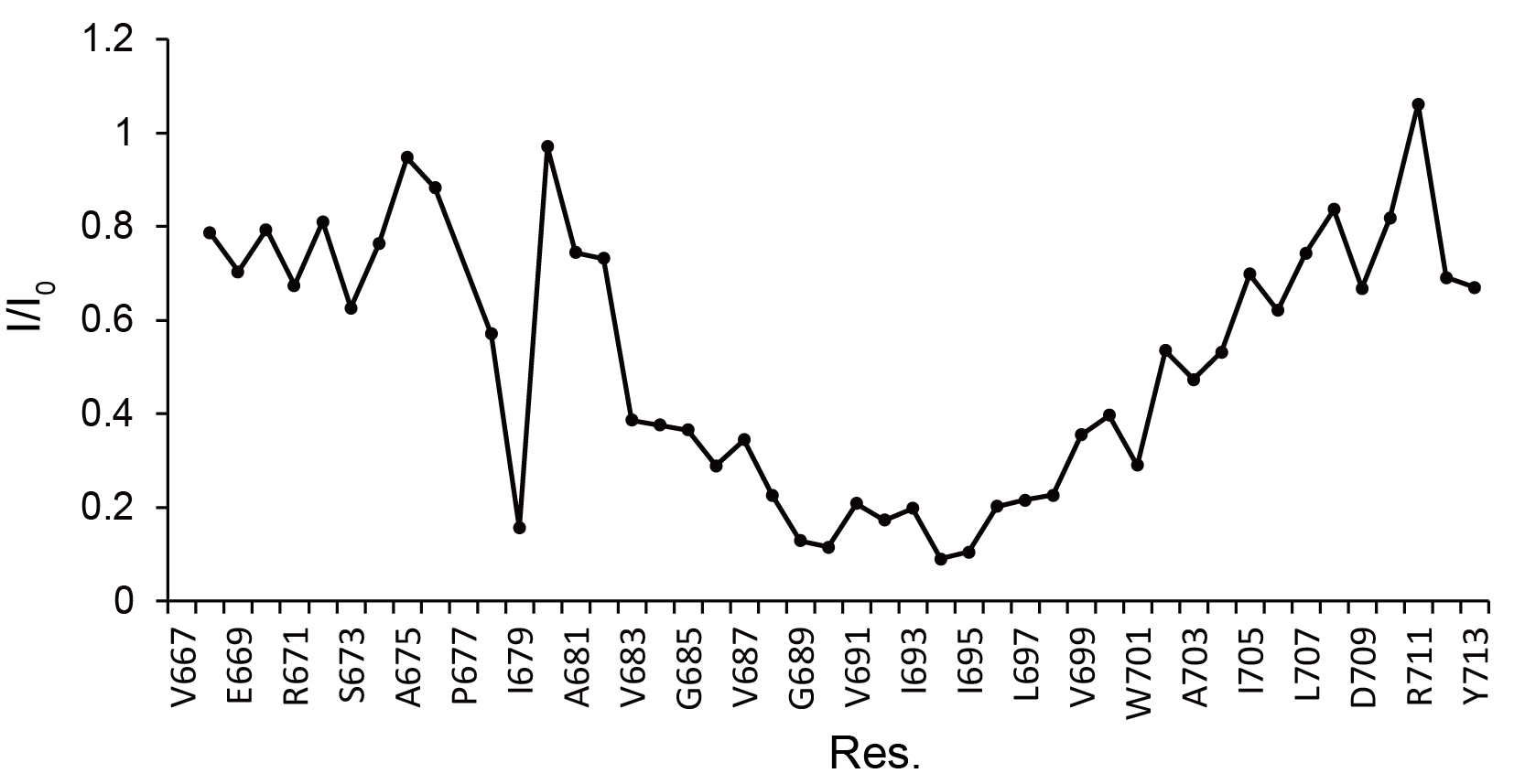

Supplement: S1 Fig — Peak intensity changes induced by the addition of 5 mM 16-DSA on integrin β2 TMD. I/I0 of 15N TROSY was used to quantify the paramagnetic effect. I represents the signal intensity with 16-DSA, while I0 represents the signal intensity without 16-DSA. The underlying data can be found in http://dx.doi.org/10.17632/tg2622h9dd.1. 15N, nitrogen-15; 16-DSA, 16-doxyl stearic acid; I/I0, intensity ratio; TMD, transmembrane domain. (TIF) [file pbio.2006525.s002.tif]

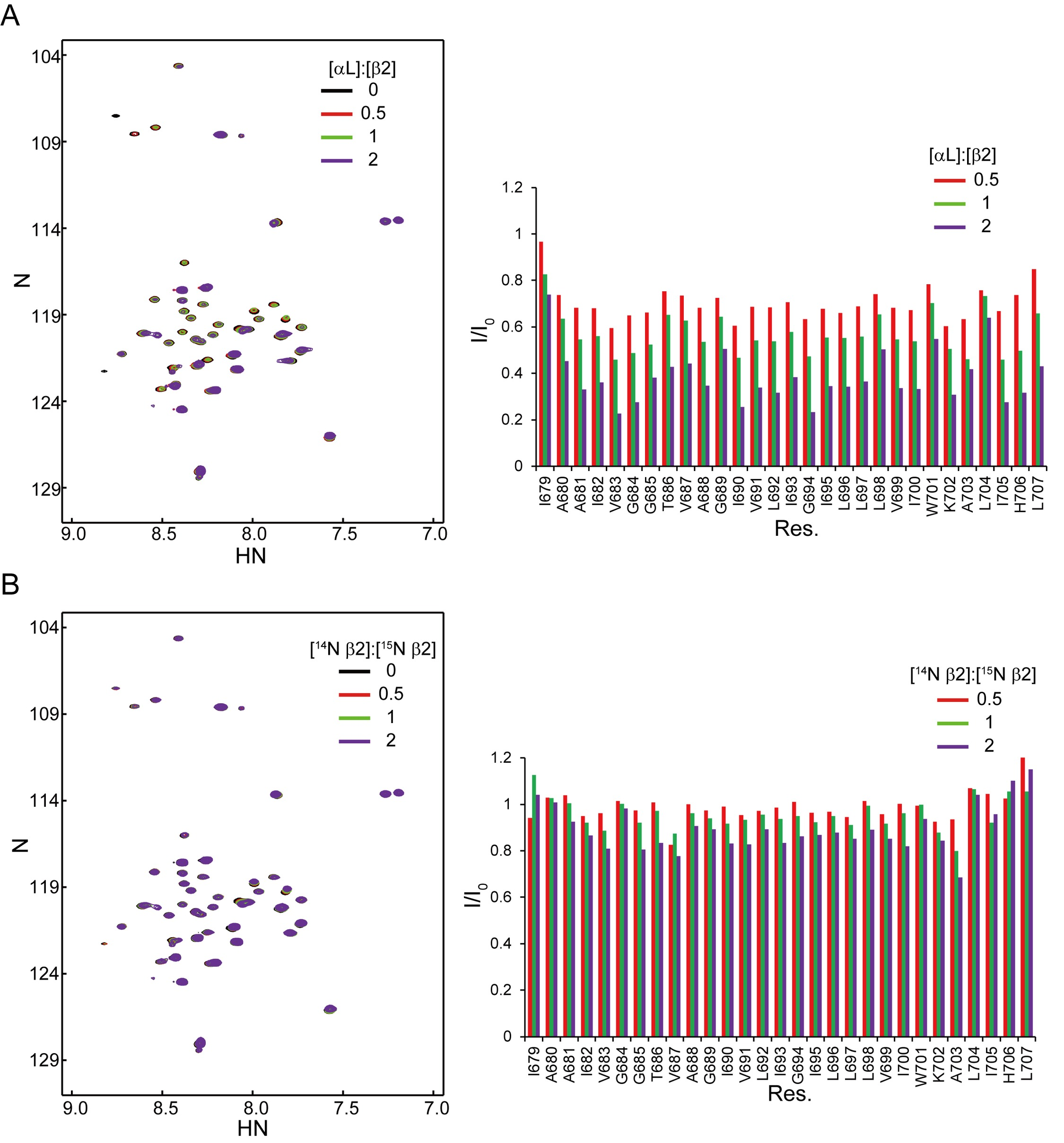

Supplement: S2 Fig — 15N-labeled β2-WT was mixed with different concentration of unlabeled αL (A) or β2 (B) in the mixture lipid bicelles. The β2-WT monomer contained 0.6 mM 15N-labeled β2 TMD peptide, 20 mM Bis-Tris (pH 6.7), 240 mM DHPC, 24 mM POPG, and 48 mM POPC. Superimposed 1H-15N TROSY-HSQC spectra of 15N-labeled β2-WT in the presence or absence of different concentrations of unlabeled αL (A) or β2 (B) are shown on left. Signal intensity comparisons of β2 TMD residues are shown on the right. I represents the signal intensity of β2 TMD residues with unlabeled αL or β2, while I0 represents the corresponding one without unlabeled αL or β2. The underlying data can be found in http://dx.doi.org/10.17632/tg2622h9dd.1. 1H, hydrogen-1, 15N, nitrogen-15; HSQC, heteronuclear single quantum coherence; POPC, 1-palmitoyl-2-oleoyl-glycero-3-phosphocholine; POPG, 1-palmitoyl-2-oleoyl-sn-glycero-3-phospho-(1'-rac-glycerol); TMD, transmembrane domain; TROSY, transverse relaxation-optimized spectroscopy; WT, wild type. (TIF) [file pbio.2006525.s003.tif]

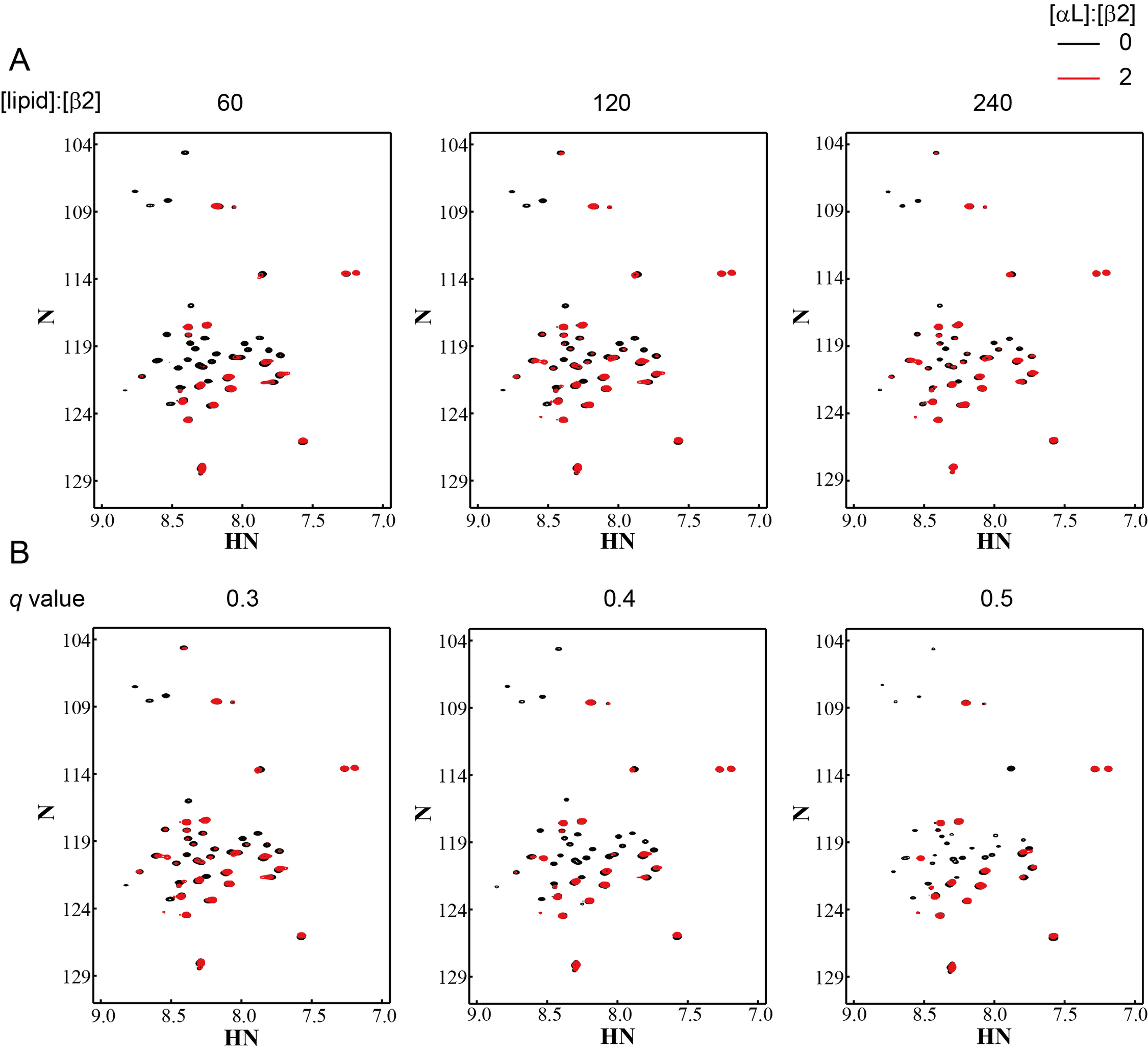

Supplement: S3 Fig — 15N-labeled β2-WT was mixed with unlabeled αL in different concentration (total lipid concentration ranging from 36 mM to 144 mM) (A) or size (q value ranging from 0.3 to 0.5) (B) of mixture lipid bicelles to form heterodimer. The β2-WT monomer contained 0.6 mM 15N-labled β2 TMD peptide. In the αLβ2 transmembrane heterodimer sample, additional 1.2 mM αL peptide was reconstituted into the bicelles. 15N, nitrogen-15; TMD, transmembrane domain; WT, wild type. (TIF) [file pbio.2006525.s004.tif]

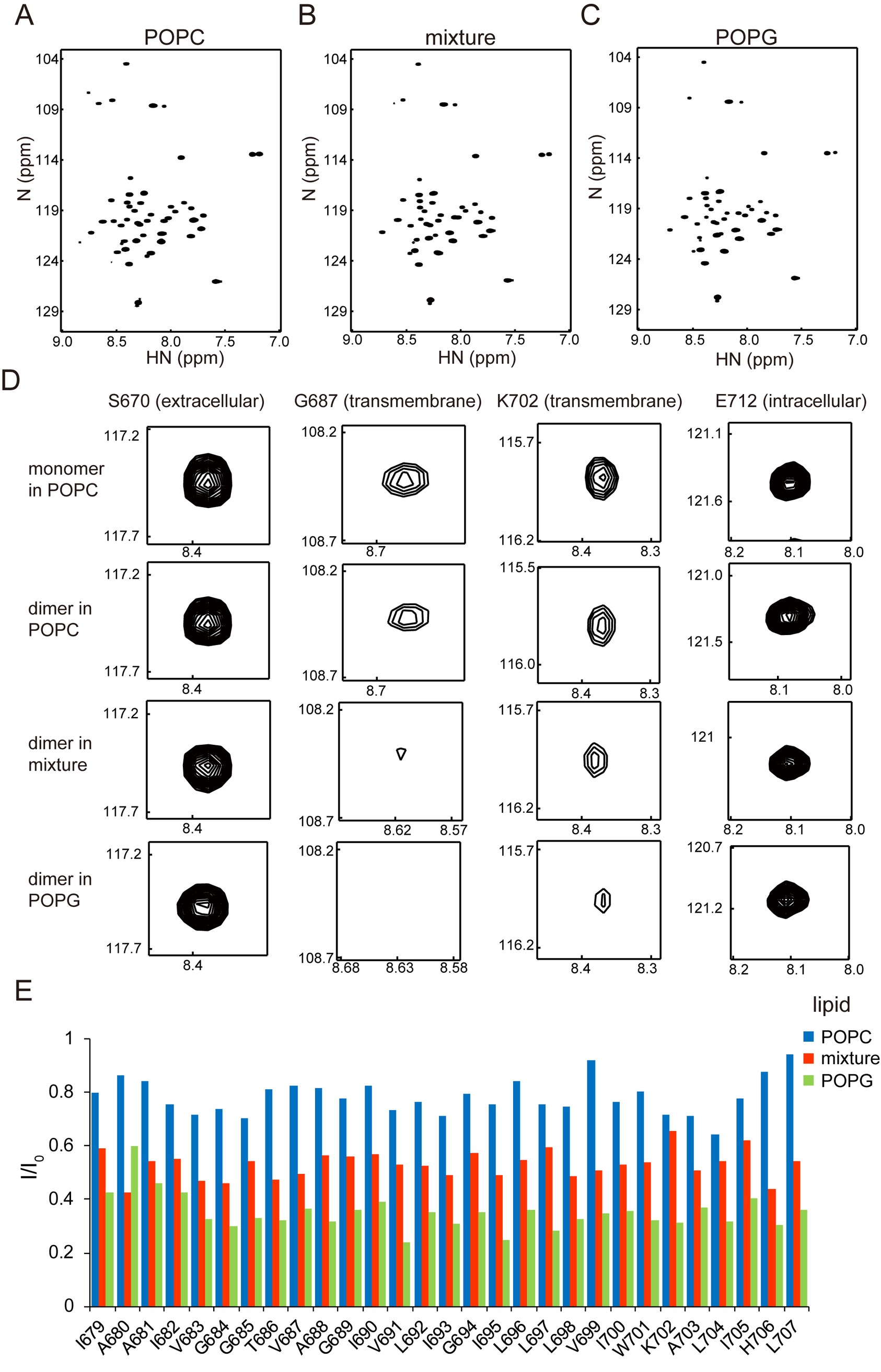

Supplement: S4 Fig — 1H-15N TROSY-HSQC spectra of αLβ2 dimer reconstituted in POPC, mixture (33% POPG, 67% POPC), or POPG bicelles. β2 was labeled by 13C/15N and αL was unlabeled. The full spectra are shown in (A–C) and representative residues from extracellular domain, transmembrane domain, and cytoplasmic domain are shown in (D). Signal intensity reductions of β2 TMD residues upon dimer formation in different lipid bicelles are shown I(E). I represented the signal intensity of β2 TMD residue in the dimer sample, while I0 represented the corresponding one in the monomer sample. The underlying data can be found in http://dx.doi.org/10.17632/tg2622h9dd.1. 1H, hydrogen-1; 15N, nitrogen-15; HSQC, heteronuclear single quantum coherence; POPC, 1-palmitoyl-2-oleoyl-glycero-3-phosphocholine; POPG, 1-palmitoyl-2-oleoyl-sn-glycero-3-phospho-(1'-rac-glycerol); TMD, transmembrane domain; TROSY, transverse relaxation-optimized spectroscopy. (TIF) [file pbio.2006525.s005.tif]

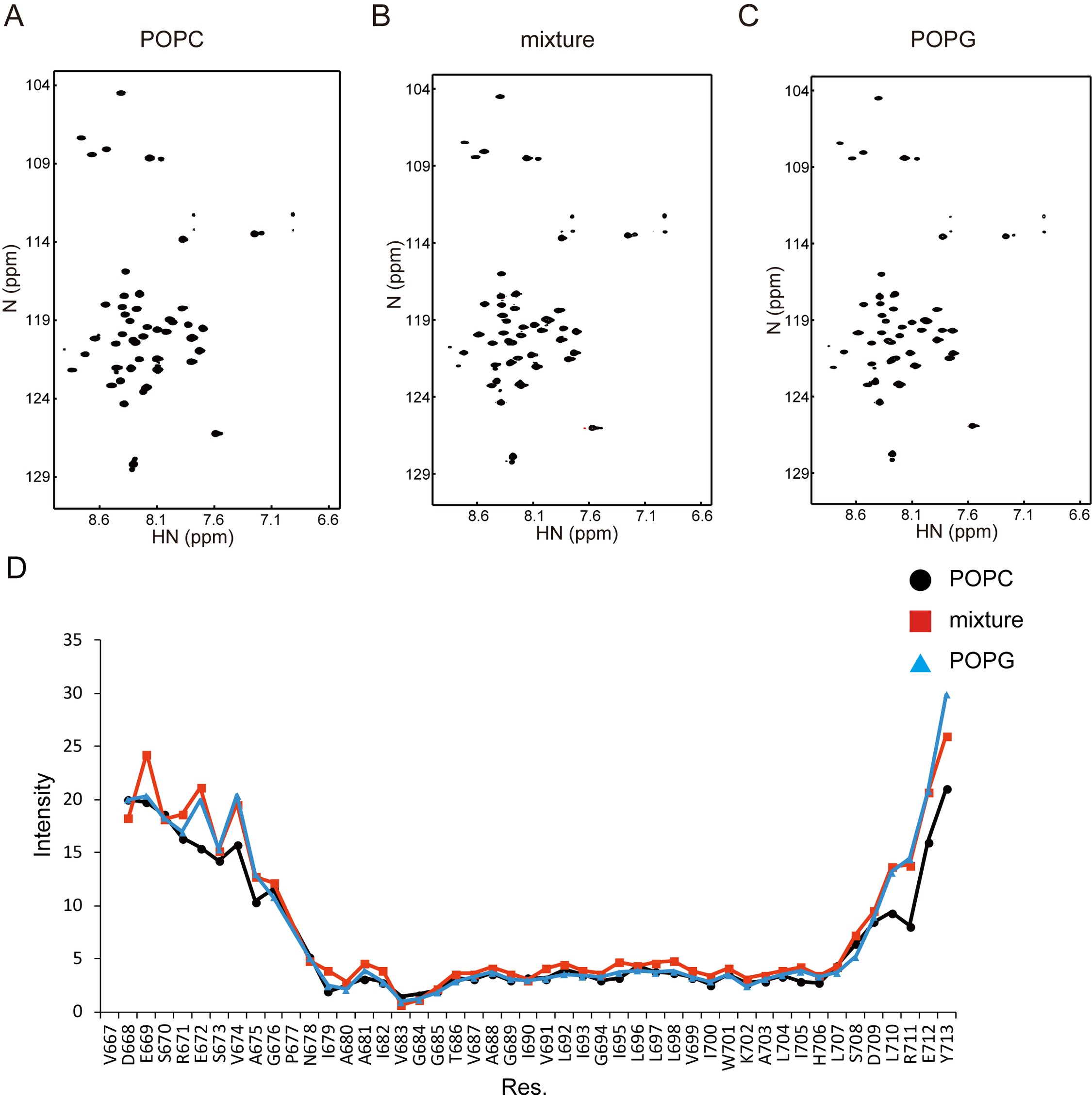

Supplement: S5 Fig — (A) 1H-15N TROSY-HSQC spectra of β2 monomer reconstituted in different lipid bicelles, i.e., POPC mixture (33% POPG, 67% POPC) or POPG bicelles. (B) Peak intensity of β2 residues in different lipid bicelles. The underlying data can be found in http://dx.doi.org/10.17632/tg2622h9dd.1. 1H, hydrogen-1; 15N, nitrogen-15; HSQC, heteronuclear single quantum coherence; POPC, 1-palmitoyl-2-oleoyl-glycero-3-phosphocholine; POPG, 1-palmitoyl-2-oleoyl-sn-glycero-3-phospho-(1'-rac-glycerol); TMD, transmembrane domain; TROSY, transverse relaxation-optimized spectroscopy. (TIF) [file pbio.2006525.s006.tif]

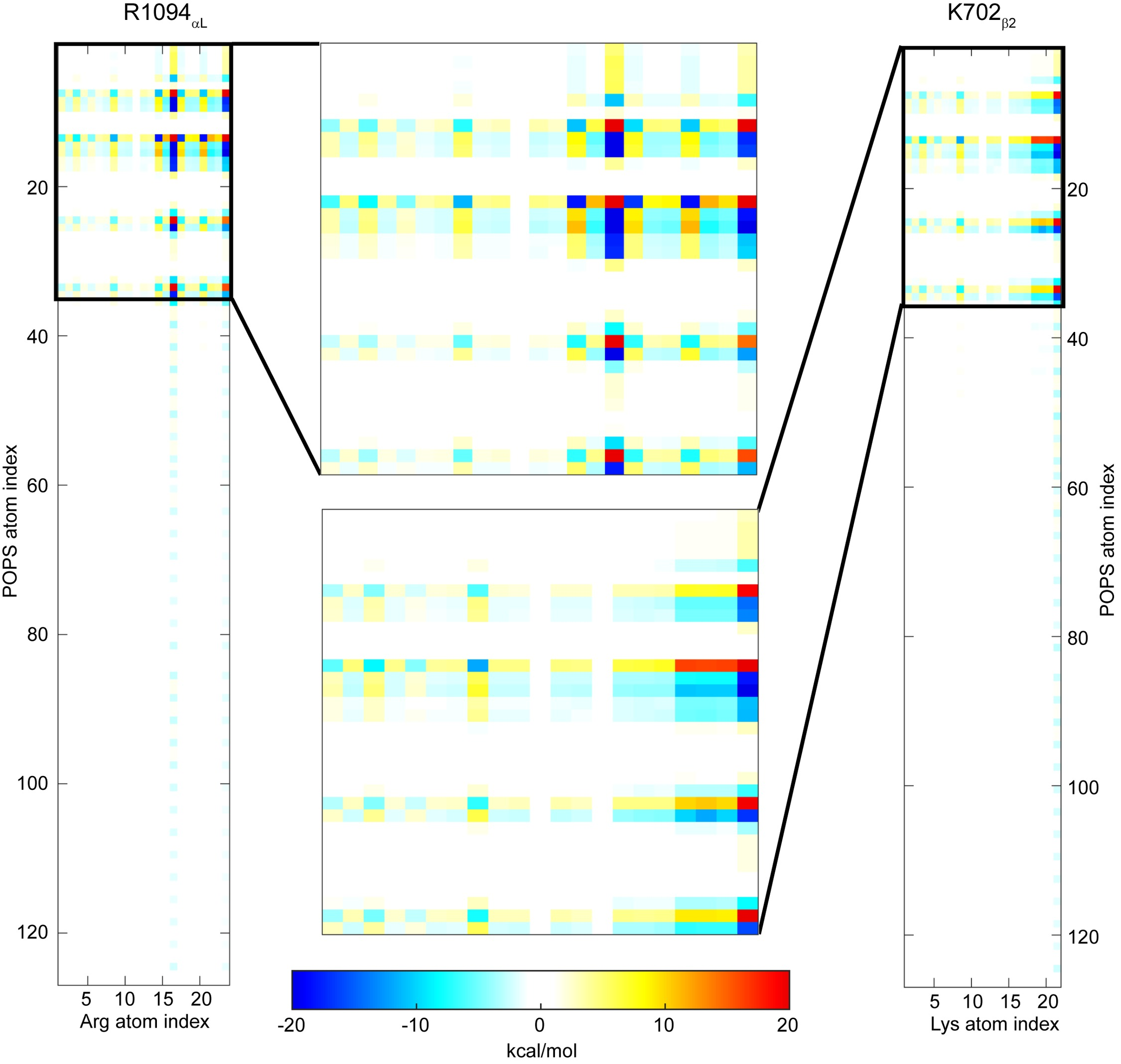

Supplement: S6 Fig — The Y-axis indicates the atom index of POPS. The x-axis indicates the atom index of αL-R1094 (left) or β2-K702 (right). The values of the interaction energy, in the range of [−20, 20], are shown as blue (negative) and red (positive) according to the color bar. The interaction energy of first 35 atoms of POPS with αL-R1094 or β2-K702 are enlarged and shown in the middle. The interaction pairs with the lowest interaction energy are highlighted by rounded rectangle in red color. POPS, 1-palmitoyl-2-oleoyl-sn-glycero-3-phospho-L-serine. (TIF) [file pbio.2006525.s007.tif]

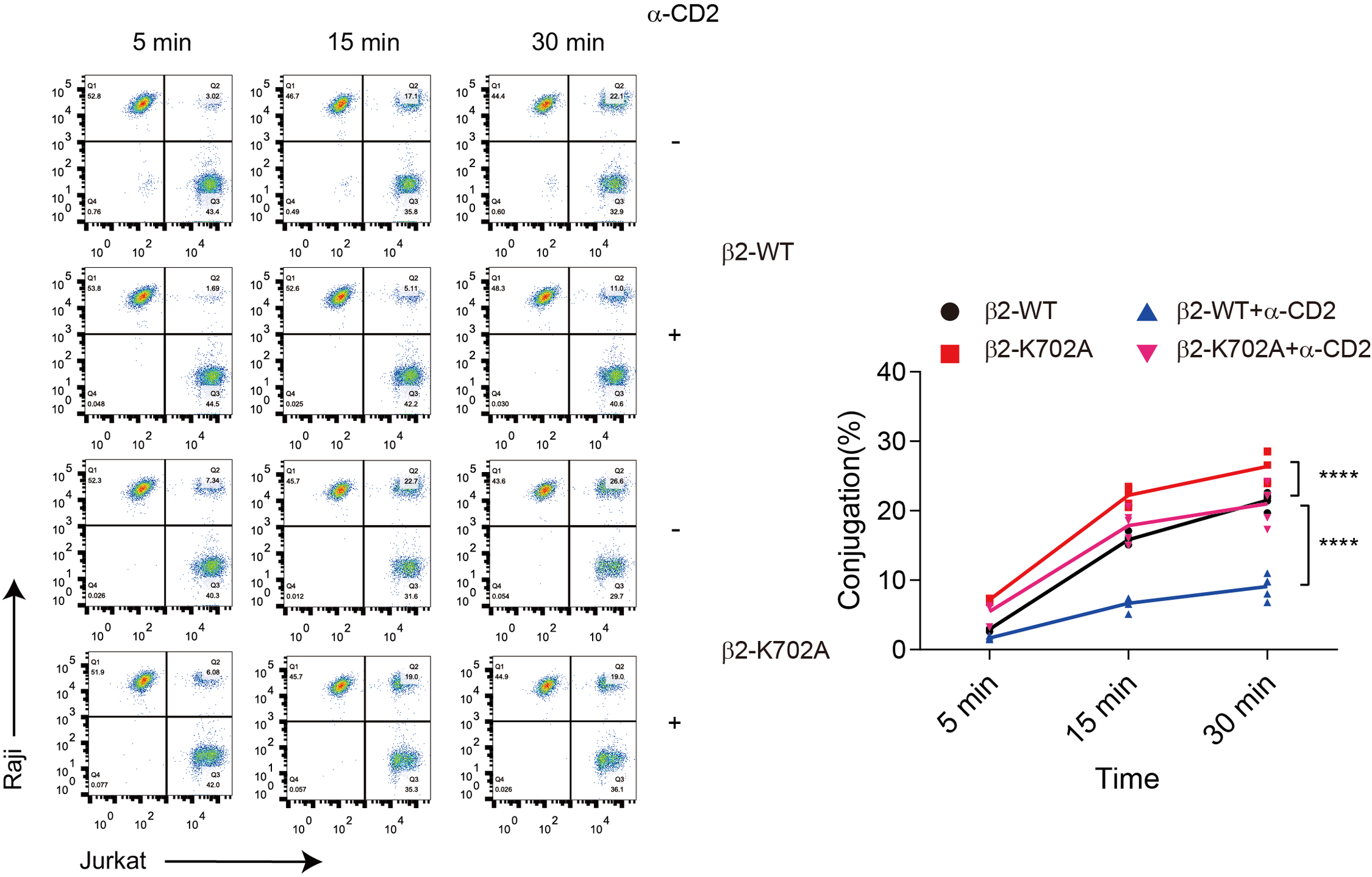

Supplement: S7 Fig — T-cell adhesion to target cells was measured by flow cytometry. Jurkat T cells and Raji B cells were labeled with Cell Tracker CSFE and Cell Tracker Deep Red, respectively. To block CD2-CD58 interaction, Jurkat T cells were pretreated with 10 μg/ml α-CD2 (RPA-2.10). Representative FACS pictures are shown at the left. The conjugates appear at the right upper corner. The underlying data can be found in http://dx.doi.org/10.17632/tg2622h9dd.1. Two-way ANOVA was used to compare the differences between β2-WT and β2-K702A in different time points (n = 5 for each group). Data are representative of three independent experiments and displayed as individual points. ****P < 0.0001. APC, antigen presenting cell; CD, cytoplasmic domain; CFSE, 5-(and-6)-Carboxyfluorescein Diacetate, Succinimidyl Ester; FACS, fluorescence-activated cell sorting; WT, wild type. (TIF) [file pbio.2006525.s008.tif]

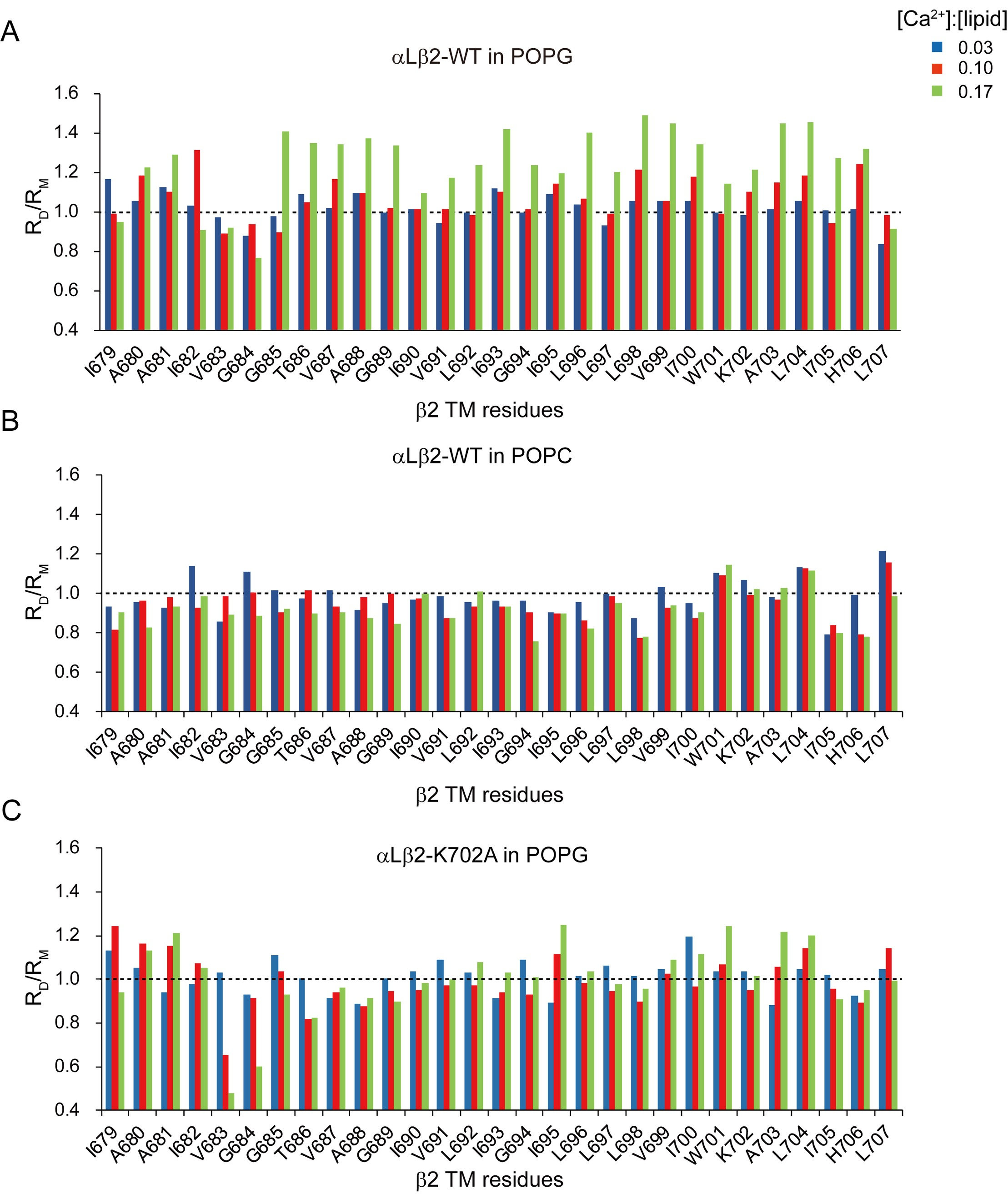

Supplement: S8 Fig — Peak intensity changes of each β2 TMD residue under Ca2+ titration are displayed as a bar graph. RD/RM values of αLβ2-WT in POPG (A), POPC (B), and αLβ2-K702A in POPG (C) are shown. RD represents ICa2+/I0Ca2+ in the dimer sample, while RM represents that ratio in the monomer sample. Ca2+:phospholipid (POPC or POPG) was from 0.03 to 0.17. The underlying data can be found in http://dx.doi.org/10.17632/tg2622h9dd.1. Ca2+, calcium ion; I0Ca2+, intensity under no Ca2+ condition; ICa2+, intensity under Ca2+ condition; POPC, 1-palmitoyl-2-oleoyl-glycero-3-phosphocholine; POPG, 1-palmitoyl-2-oleoyl-sn-glycero-3-phospho-(1'-rac-glycerol); TMD, transmembrane domain; WT, wild type. (TIF) [file pbio.2006525.s009.tif]

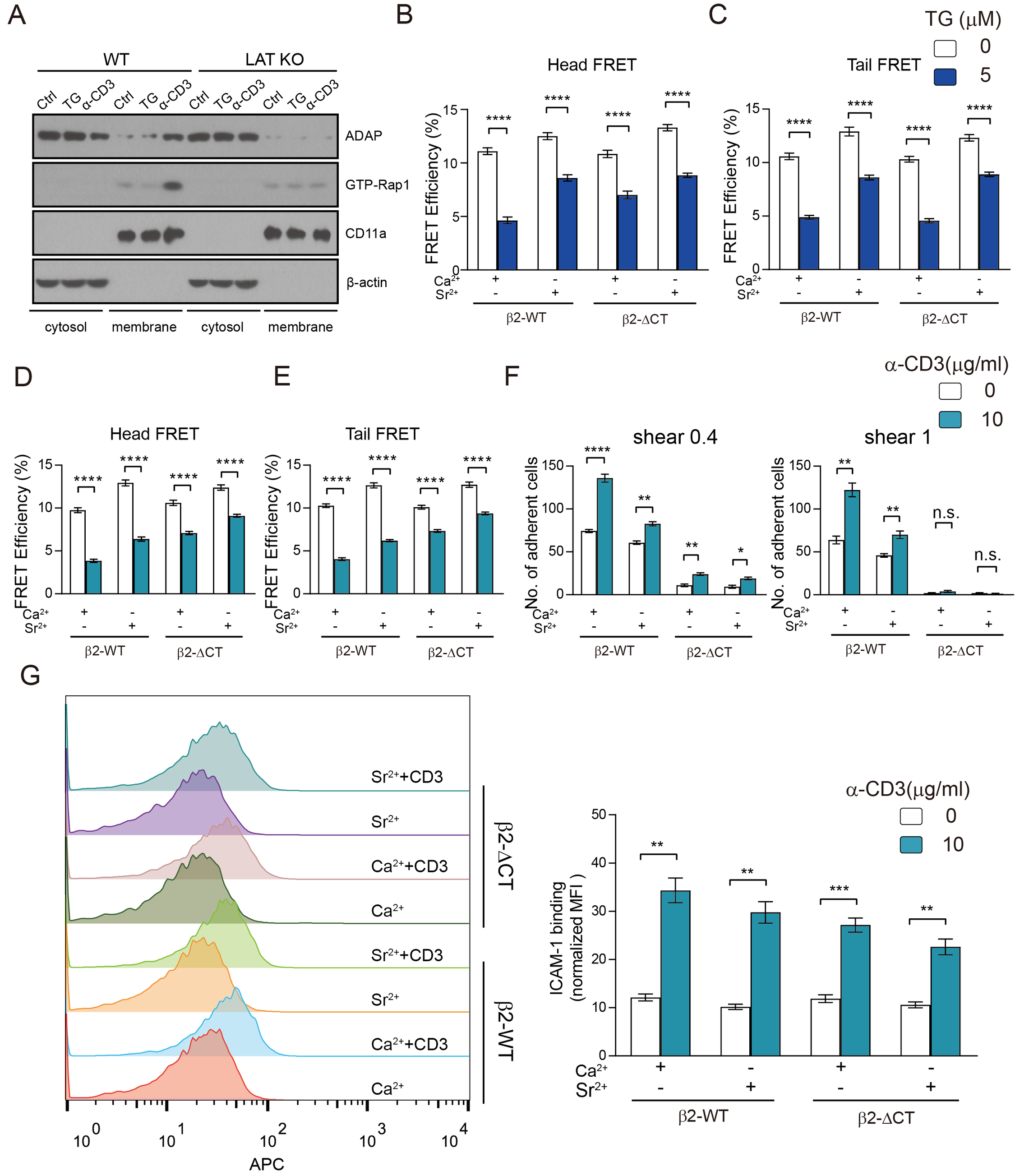

Supplement: S9 Fig — (A) Sr2+ does not cause membrane recruitment of ADAP and Rap1. Western blot analysis of ADAP and GTP-Rap1 recruitment to plasma membrane in WT and LAT-KO Jurkat T cells. Cells were either left unstimulated or stimulated with 5μM TG or 10 μg/ml α-CD3ε (UCHT-1) in HBSS containing 5 mM Sr2+/1 mM Mg2+ for 5 min and subjected to cytosolic and plasma membrane fractionation. Active Rap1 (GTP-Rap1) was isolated using a GST-RalGDS-Rap1 binding domain fusion protein. To control the fractionation efficiency, fractions were assessed for the presence of CD11a and β-actin. (B–E) β2-KO Jurkat cells were reconstituted with β2-WT, cytoplasmic domain truncation mutant. WT or cytoplasmic domain truncation mutant (ΔCT) αLβ2 conformational changes induced by TG (B, C) or TCR (D, E) stimulation were measured by the Head and Tail FRET assays. (F) Adhesive modality of Jurkat T cells expressing WT or ΔCT mutant αLβ2 on ICAM-1 substrates at a wall shear stress of 0.4 dyn/cm2 (left panel) and 1 dyn/cm2 (right panel). (G) Binding of soluble ICAM-1 to Jurkat T cells expressing WT or ΔCT mutant αLβ2 treated with or without 10 μg/ml α-CD3ε (UCHT-1) in HBSS containing 1 mM Ca2+/ Mg2+ or 5 mM Sr2+/1 mM Mg2+. ICAM-1 binding was measured by flow cytometry and presented as MFI normalized to integrin expression (TS1/18 binding). The underlying data of panel B–G can be found in http://dx.doi.org/10.17632/tg2622h9dd.1. Data are representative of two independent experiments and displayed as mean ± SEM. Student t test was used to analyze the differences between two groups. *P < 0.05; **P < 0.01, ***P < 0.001, ****P < 0.0001. ADAP, adhesion and degranulation-promoting adaptor protein; Ca2+,calcium ion; CD, cytoplasmic domain; FRET, fluorescence resonance energy transfer; HBSS, Hank’s Balanced Salt Solution; ICAM-1, intercellular adhesion molecule 1; MFI, mean fluorescence intensity; Mg2+, magnesium ion; n.s., not significant; Sr2+, strontium ion; TCR, T-cell receptor; TG, thapsigargin; WT, wild type. (TIF) [file pbio.2006525.s010.tif]

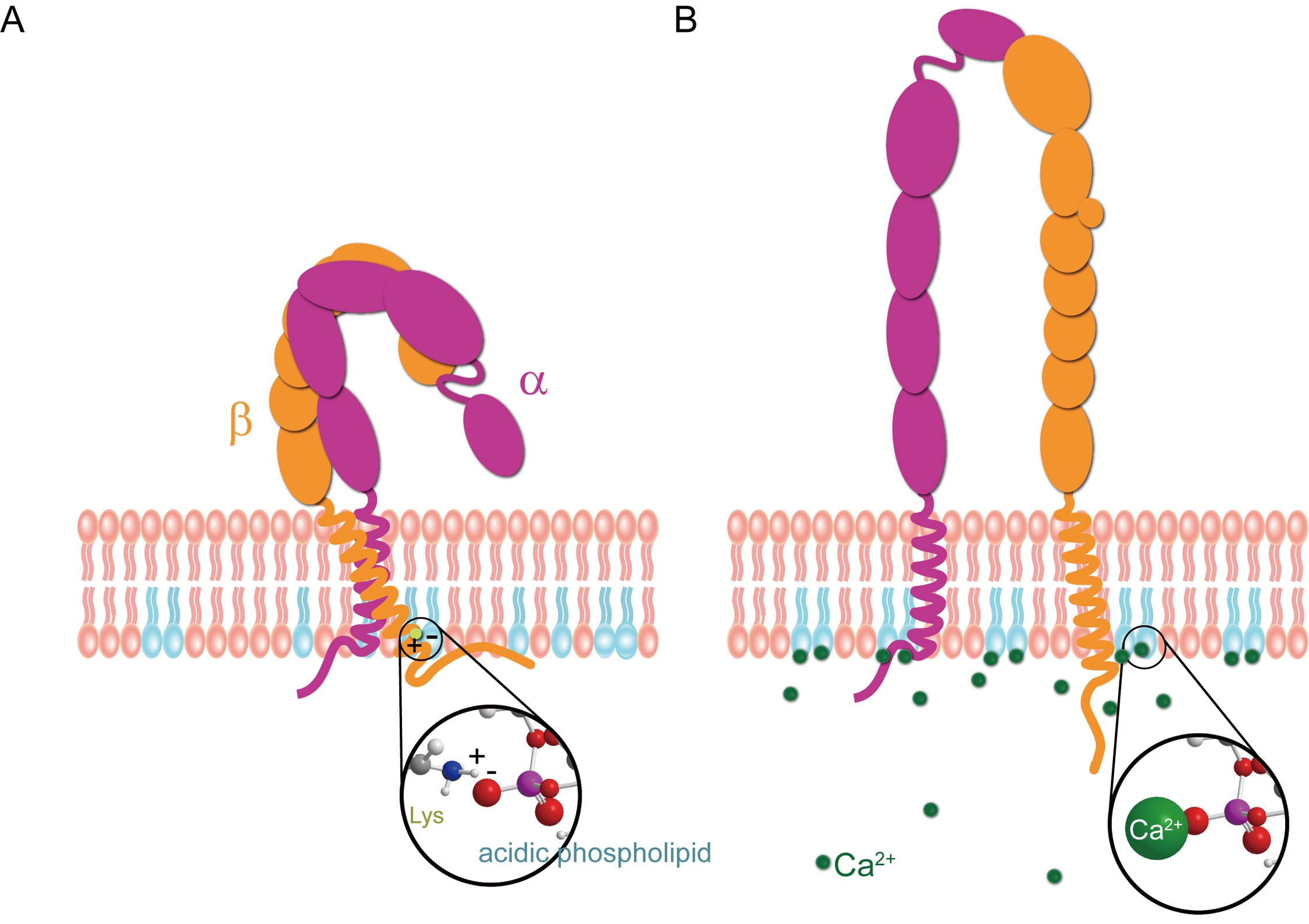

Supplement: S10 Fig — (A) In resting T cells, the ionic interaction between the β2-K702 amino group and the phosphate group of acidic phospholipids stabilizes transmembrane association between αL and β2 subunits, thus keeping αLβ2 in low-affinity conformation. (B) In activated T cells, Ca2+ ions quickly influx and generate high local [Ca2+] [5, 7]. Local Ca2+ ions can directly neutralize the lipid phosphate group to destabilize αLβ2 transmembrane association, thus turning αLβ2 to high-affinity conformation. This effect is independent of Ca2+ downstream signaling and integrin inside-out signaling. Ca2+, calcium ion. (TIF) [file pbio.2006525.s011.tif]
